# Fractionation of High-Value Compounds from Hops Using an Optimised Sequential Extraction Procedure

**DOI:** 10.3390/antiox13010045

**Published:** 2023-12-26

**Authors:** Ana I. Paniagua-García, David Ruano-Rosa, Rebeca Díez-Antolínez

**Affiliations:** 1 Centre of Biofuels and Bioproducts, Agricultural Technological Institute of Castilla y León, Villarejo de Órbigo, E-24358 León, Spain; dieantre@itacyl.es; 2 Agricultural Technological Institute of Castilla y León, Ctra. De Burgos, Km 119, E-47071 Valladolid, Spain; david.ruano@juntadeandalucia.es

**Keywords:** hops, sequential extraction, hop bitter acids, xanthohumol, phenolic compounds, antioxidant activity

## Abstract

This study describes the development and optimisation of a process for the extraction and fractionation of high-value compounds from hops. Firstly, the efficacy of ten organic solvents was compared for performing the initial solid–liquid extraction of compounds from hop pellets with subsequent fractionation steps. A methanol–dichloromethane mixture was selected and the extraction variables were optimised in order to maximise the recovery of valuable hop compounds separated into different streams (α- and β-acids in soft resins, xanthohumol in hard resins, and phenolics in spent solids) after fractionation steps. The optimisation results showed that extraction of hop pellets performed at room temperature with 19.7% (*v*/*v*) methanol for 89 min yielded recoveries of 86.57% α-acids and 89.14% β-acids in soft resins, 78.48% xanthohumol in hard resins and 67.10% phenolics in spent solids. These conditions were successfully validated using six hop varieties. Moreover, the antioxidant properties of all recovered fractions were compared and the soft resins showed the highest antioxidant activities, reaching values of 3.91 ± 0.10 g AAE/100 g for ferric reducing power (FRAP) and 0.10 ± 0.01 mg/mL for 50% of radical scavenging activity (EC50). The optimised sequential extraction could serve as a basis for larger scale-up for industrial production.

## 1. Introduction

In recent years, there has been growing concern about the misuse of synthetic chemical products which are often associated with health problems in humans, animals and the environment [1]. Natural bioactive phytocompounds, derived from an ever-increasing number of different plants and plant by-products, have attracted the attention of industry due to their wide range of applications. Furthermore, these biocompounds are considered as eco-friendly, natural and generally safe products. In this context, several studies using in vitro and in vivo models have shown that the hop plant (*Humulus lupulus* L.) has numerous biological activities and has been used for its antimicrobial, antioxidant, sedative, anti-inflammatory and phytoestrogenic properties and as a brewing ingredient [2].

Hop is a long-lived dioecious perennial climbing plant of the Cannabaceae family [3]. Although the plant is native to Europe, western Asia and North America, it is mostly restricted to latitudes between 35° and 55° due to its need for moderate temperatures and rainfall [3]. Only female plants produce the inflorescence, known as strobiles, cones or hops, where the lupulin glands are found [2]. The lupulin glands contain essential oils and resins [4]. The essential oils are composed of terpenes, carboxylic acids, esters, aldehydes and ketones and the profile of aromatic compounds differs between the phenophases of the varieties [5]. Total hop resins are defined as the fraction soluble in diethyl ether and cold methanol and can be divided into soft and hard resins based on their solubility in different solvents [6]. Soft resins are soluble in hexane and their main compounds are α-acids (cohumulone, humulone and adhumulone) and β-acids (colupulone, lupulone and adlupulone), which are the main source of flavour and bitterness of hops and have antimicrobial properties [6]. The total resins fraction of hops, which is insoluble in hexane and soluble in methanol and diethyl ether, is called hard resins [6]. The main compound found in hard resins is xanthohumol (a prenylated flavonoid found only in hop inflorescences) [6]. Interest in xanthohumol has increased in recent years due to its antioxidant, antimicrobial, antiviral, antifungal, anti-inflammatory, anticancer, antiplasmodial and anti-obesity activities [7]. In addition to essential oils and resins, phenolics are another type of compounds of great industrial interest [4]. Hop phenolic compounds mainly consist of flavonol derivatives (particularly quercetin and kaempferol glycosides), flavan-3-ols (catechin, epicatechin, tannins and proanthocyanidins), phenolic carboxylic acids (especially chlorogenic acid and its isomers, coumaroylquinic acids and feruloylquinic acids) and other polyphenols (prenylflavonoids, resveratrol, multifidols) [6,8].

In recent decades, knowledge of the chemical structures and reactivity of valuable hop compounds has increased the production and use of hop extracts. Moreover, hop extracts protect biocompounds from oxidation and allow a better dosage. Nevertheless, it is imperative to develop sustainable and efficient extraction techniques that guarantee the quality of the extracts obtained from hops [9]. To extract biocompounds from plant material, solid–liquid (S-L) extraction techniques using organic solvents followed by solvent distillation are the most widely used [2]. The efficiency of extraction techniques depends on the solvent used. Considering industrial applications, the choice of organic solvents is limited to non-toxic solvents. The maximum residue levels of several solvents in food products are strictly defined by Directive 2009/32/EC [10]. Thus, for the extraction of hop-derived resins, alcohols, ketones, hexane, methylene chloride, trichloroethylene and chloroform have been used [5]. For phenolic compounds, the most commonly used extraction solvents are ethanol, methanol and water [9].

New extraction technologies have been developed and applied for hop, including supercritical CO_2_ extraction (sc-CO_2_), microwave-assisted extraction (MAE), ultrasound-assisted extraction (UAE), and accelerated solvent extraction (ASE). These technologies are highly efficient, reduce the use of solvents and water and allow the valorisation of the residues obtained after extraction [11]. Moreover, sc-CO_2_ offers a number of additional advantages, such as the absence of undesirable organic solvent residues in the final extract, lower environmental impact, oxidation-free products as extraction is performed in an oxygen-free atmosphere and a simple scalable process [5]. Thus, sc-CO_2_ is currently widely used to produce hop extracts with high levels of essential oils and bitter acids [12]. However, the main limitation of sc-CO_2_ is its low ability to dissolve polar compounds, and therefore the addition of polar co-solvents is required to increase the extraction yield of these types of compounds [2]. For MAE, UAE and ASE, the main constraints are related to the adaptation of these techniques to industry, which has major drawbacks such as the need to update infrastructures and the difficulty of the scale-up [11].

Several previous investigations have attributed the health benefits of hops to the antioxidant activity and hydroxyl radical scavenging of α-acids, β-acids, xanthohumol and phenolic compounds [13]. Despite the abundance of the literature on solvents and extraction techniques of valuable compounds from different plant materials, there are few studies on the effect of extraction conditions on the content of bitter acids and phenolic compounds, or on the antioxidant activities of hops and their products [9]. Furthermore, only some hop varieties and industrial extracts including plugs and pellets have been studied [14]. Moreover, to the best of our knowledge, no studies have been found in the literature focusing on the optimisation of a sequential extraction process to recover the different high-value compounds of hops (bitter acids, xanthohumol and phenolic compounds) separated into different streams, in order to simultaneously maximise their recovery yields.

In this research, the efficacy of ten organic solvents was compared for performing an initial S-L extraction of compounds from hop pellets followed by sequential fractionation. This approach is easily adaptable to an industrial scale and allows the recovery of the valuable hop compounds separated into different streams (α- and β-acids in soft resins, xanthohumol in hard resins and phenolic compounds in spent solids). The objectives of this work were: (i) to optimise the S-L extraction conditions (solvent/s composition and stirring time) in order to simultaneously maximise the yield of the main valuable hop compounds separated into different fractions, (ii) to verify the effectiveness of the optimal extraction conditions by applying them to six hop varieties (pellet form) and (iii) to determine the chemical composition and assess the efficiency of 2,2-diphenyl-1-picrylhydrazine (DPPH) and ferric reducing antioxidant power (FRAP) assays of all the streams recovered from the six hop varieties.

## 2. Materials and Methods

### 2.1. Reagents and Biomasses

Analytical grade methanol, ethyl acetate, diethyl ether and formaldehyde and HPLC grade methanol and dichloromethane were provided by Thermo Fisher Scientific (Waltham, MA, USA). Analytical grade ethanol (96%) and Folin–Ciocalteau reagent were obtained from Scharlab (Sentmenat, Spain). Analytical grade hexane, sodium hydroxide and glacial acetic acid were supplied from Panreac (Castellar del Vallès, Spain). Analytical grade ferric chloride hexahydrate was purchased from VWR Chemicals (Radnor, PA, USA). Analytical grade acetone, sodium carbonate, sodium acetate, hydrochloric acid (37%), 2,2-diphenyl-1-picrylhydrazyl (DPPH), 2,4,6-tris(2-pyridyl)-s-triazine (TPTZ), HPLC grade orthophosphoric acid (85%) and the following analytical standards: gallic acid monohydrate (100.6%), (+)-catechin hydrate (≥96%), ascorbic acid (≥99%), xanthohumol (≥96%), (-)-β-pinene (99.6%), myrcene (91.7%), S-(-)-limonene (97.9%), linalool (97.0%), geraniol (99.1%), 2-undecanone (99.1%), β-caryophyllene (99.5%), β-farnesene (≥90.0%) and α-humulene (96.8%) were purchased from Sigma-Aldrich (Steinheim, Germany). Bitter acids mixture standard (international calibration extract ICE-4) was provided by Labor Veritas Co. (Zürich, Switzerland). ICE-4 was reported to contain 42.58% α-acids (10.98% cohumulone and 31.60% n+adhumulone) and 26.54% β-acids (13.02% colupulone and 13.52% n+adlupulone). Deionized water (resistivity > 18 MΩ/cm) was produced by using a Milli-Q ultrapure system from Millipore (Burlington, MA, USA).

Six different hop varieties (Nugget, Columbus, Cascade, Eureka, Chinook and Fuggle), in dried pellet form with a moisture content between 6 and 10% (*w*/*w*), were kindly supplied by Órbigo Valley S.L. (Villamor de Órbigo, León, Spain). Each hop pellet sample was packaged in a plastic bag, sealed under vacuum and stored in the dark at room temperature until use. Prior to analysis, the hop pellets were ground to fine powder in a SM100 Comfort rotatory mill (Retsch GmbH, Haan, Germany) and kept protected from light and humidity until analysis.

### 2.2. Development of a Sequential Extraction Method to Recover High-Value Compounds from Hops

In order to increase the efficiency of the extraction of high-value compounds from hops and their subsequent separation in different streams, several organic solvents were compared in extraction experiments. Therein, extractions were carried out on 20 g of crushed hop pellets (Nugget variety) placed in 1 L Erlenmeyer flasks and extracted with 380 g of one of the following organic solvents: methanol, ethanol, dichloromethane, acetone, ethyl acetate, hexane, methanol–diethyl ether mixture 25:75 and 50:50 (*v*/*v*) and methanol–dichloromethane mixture 25:75 and 50:50 (*v*/*v*). The flasks were capped with a rubber septum and each preparation was left under magnetic stirring at room temperature for 1 h. Each extraction experiment was performed in triplicate.

After the S-L extraction, the liquid extract (with a high concentration of α-acids, β-acids and xanthohumol) was recovered by vacuum filtration using a Büchner funnel with cellulose filters (20–25 µm, Model 1238, Filter Lab, Barcelona, Spain). The spent solid was washed with two aliquots of 40 mL of the same solvent used for the S-L extraction which were added to the liquid extract. Then, the solid (with a high content of phenolic compounds) was dried in an oven at 45 °C for 48 h, weighed and stored until analysis to determine its chemical composition. The recovered organic solvent was removed by rotary evaporation using a Büchi R-215 rotavapor (Flawil, Switzerland) and the residue remaining in the flask (total resins) was weighed. In order to separate two different fractions contained in the total resins, extraction with hexane was carried out. For this, the flask containing the total resins was filled with 200 mL of hexane, tapped with a rubber cup and shaken vigorously for 2 min. Then, the mixture was filtered at atmospheric pressure through a cellulose filter (7–9 µm, Model 1242, Filter Lab) using a stainless steel Büchner funnel. The solids retained on the filter (hard resins) and the residue obtained after hexane evaporation in a rotary evaporator (soft resins) were weighed and retained for further analysis.

**Figure 1:**
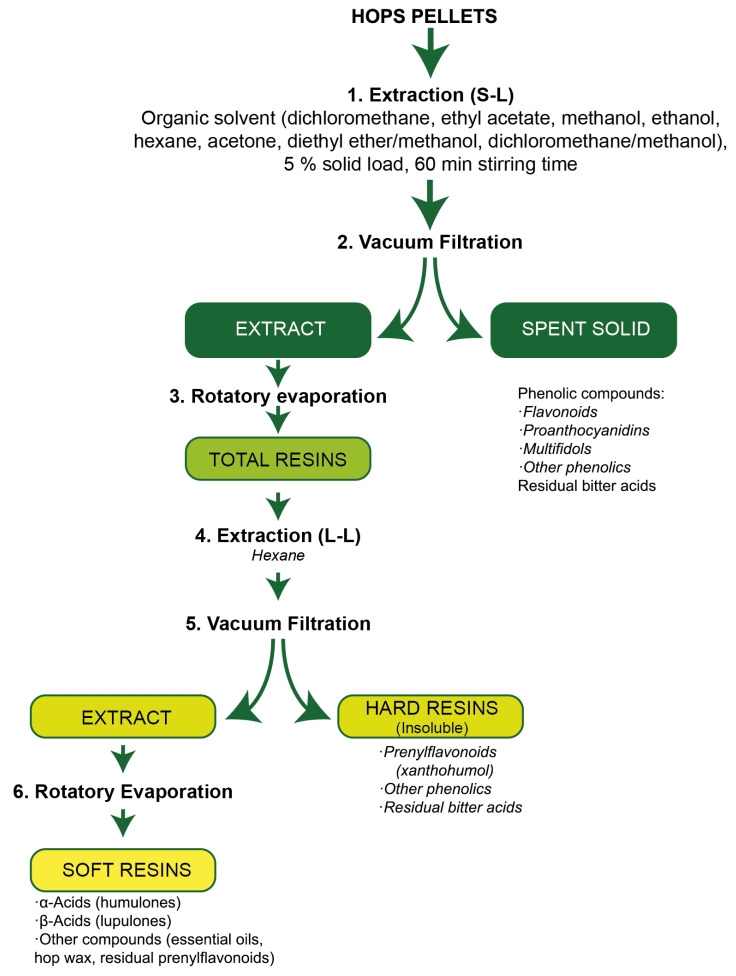
Flow diagram of the sequential extraction procedure.

In this way, three different fractions (spent solids, hard resins and soft resins) were obtained from hop pellets by applying the described sequential extraction procedure (Figure 1) and were analysed for the contents of α- acids, β-acids, xanthohumol and phenolic compounds. The recovery (%) of each compound (xanthohumol) or group of compounds (α- acids, β-acids and phenolics) in each fraction was calculated considering the corresponding contents in the raw material.

### 2.3. Optimisation of the S-L Extraction Method

For extraction of high-value compounds of hops, the methanol–dichloromethane mixture was the most efficient solvent, as it allowed higher recoveries of α- and β-acids in soft resins, xanthohumol in hard resins and phenolic compounds in spent solids (according to the procedure described in Section 2.2). 

The S-L extraction variables were then optimised using response surface methodology (RSM) to maximise the recovery yields mentioned above. A two-variable central composite rotatable design (CCRD) was used to determine the optimum combinations of methanol concentration (%, *v*/*v*) in the solvent mixture and extraction time (min). The extraction time ranged from 5 to 120 min and, based on the experiments performed, the desired range of methanol concentration was defined as 0% to 50% (dichloromethane concentration from 50% to 100%). The experimental design resulted in 13 trials (4 trials for factorial design, 4 trials for axial points and 5 trials for replication of the central point). Thus, methanol concentration was studied at three levels (7.3, 25.0 and 42.7%) with two axial points (0.0 and 50.0%) and extraction time at three levels (21.8, 62.5 and 103.2 min) with two axial points (5.0 and 120.0 min). All the experiments were carried out with crushed hop pellets of the Nugget variety. The solid load was fixed at 5% (*w*/*w*) (20 g solid + 380 g solvent) and a magnetic stirrer was used. All the experiments were performed at room temperature, as oxidation and isomerisation reactions of α-acids can occur above 40 °C [5].

The output results (recovery yields) were fitted to four second-order polynomial equations (one for each response variable) and the regression coefficients were determined using analysis of variance (ANOVA). Optimal extraction variables were calculated from the models. Further sequential extraction trials were carried out under the predicted optimal conditions to validate the regression model.

Finally, the optimal extraction variables were applied to perform the complete sequential extraction process with other hop varieties (Columbus, Cascade, Eureka, Chinook and Fuggle), also in pellet form, in order to compare the recovery of high-value compounds in the separated fractions with those recovery yields predicted by the quadratic model calculated for the Nugget variety.

### 2.4. Analytical Methods

#### 2.4.1. Moisture Content

The moisture content of the hop pellets was determined in triplicate by loss on drying in an oven at 103–105 °C for 1 h according to the European Brewery Convention (EBC) analytical method EBC 7.2 [15].

#### 2.4.2. Essential Oils Content

The essential oil content of the six hop varieties was measured after steam distillation using a Clevenger distillation apparatus following EBC method 7.10 [16]. Briefly, 100 g of crushed hop pellets were placed in a 5000 mL distillation flask, 3000 mL of deionised water was added and the mixture was distilled for 3 h. The main compounds of hop essential oils (β-pinene, myrcene, limonene, linalool, geraniol, 2-undecanone, β-caryophyllene, β-farnesene and α-humulene) were then determined by capillary gas chromatography (GC) using an Agilent 7890 GC equipped with a flame ionisation detector (FID) and an HP 5 (30 m × 0.320 mm, 0.25 µm) column (Agilent Technologies, Santa Clara, CA, USA) according to EBC method 7.12 [17]. Helium (purity 99.999%) was used as carrier gas at a flow rate of 1 mL/min. The column temperature programme began at 50 °C (held 1 min) and then increased at 3 °C/min until 260 °C (held 15 min). The injector was maintained at 200 °C and the detector at 260 °C. The injection volume was 1 µL, the split ratio was 1:50 and the run time was 86 min.

#### 2.4.3. α-Acids and β-Acids Content

The α-acids and β-acids contained in fresh hops, spent solids and resins were measured by high-performance liquid chromatography using an Agilent 1100 series HPLC system (Agilent Technologies) equipped with a G1313A autosampler, a G1311A quaternary pump, a G1316A thermostatted column and a G1315B DAD detector, following a modification of the method described in EBC 7.7 [18]. Briefly, bitter compounds were extracted from 10 g of ground hops with 20 mL of methanol, 100 mL of diethyl ether and 40 mL of 0.1 mol/L hydrochloric acid solution. An aliquot of the diethyl ether/methanol extract was then diluted with methanol and the α- and β-acids contents were determined using HPLC. In the case of hop resins (total, soft and hard resins), they were dissolved in methanol. The separation was carried out with an analytical EC Nucledur 100-5 C18 ec (125 mm × 4.0 mm, 5 µm) column (Macherey-Nagel GmbH & Co. KG, Dueren, Germany) operated at 40 °C. The mobile phase consisted of two solvents: solvent A, aqueous 4% (*v*/*v*) phosphoric acid and solvent B, methanol. The flow rate was 1.0 mL/min with 20% solvent A and 80% solvent B under isocratic conditions and a run time of 25 min. The injection volume was 20 µL and the diode array detector was set at 314 nm. All the samples were filtered through PTFE filters of 0.22 µm (Agilent Technologies) prior to the injection.

Thus, the concentrations of cohumulone, n+adhumulone, colupulone and n+adlupulone were determined and the α-acids were expressed as the sum of the humulones and the β-acids were expressed as the sum of the lupulones.

#### 2.4.4. Xanthohumol Content

The determination of the xanthohumol content in hops, resins and spent solids was performed according to EBC method 7.15 [19]. Basically, the extraction and chromatographic procedures followed were the same as for the determination of α- and β-acids content although the diode array detector was set at 370 nm.

#### 2.4.5. Total Phenolic Content

For the quantification of the total phenolic compounds (TPC) contained in hops pellets and spent solids, the extraction was carried out following EBC method 7.14 [20] with minor modifications. Briefly, 1 g of ground hop pellets or spent solids were placed in a 500 mL balloon flask, 100 mL of deionised water was added and the mixture was boiled under reflux for 20 min. Thereby, most of the phenolic compounds were extracted, with the exception of xanthohumol, which has very low solubility in water [21]. In the case of hop resins, they were dissolved in ethanol prior to analysis. TPC were determined spectrophotometrically using Folin–Ciocalteu reagent according to the procedure described by Singleton and Rossi [22] with some modifications. An aliquot of 50 µL of each aqueous extract (fresh hops or spent solid) or ethanolic solution (soft resins or hard resins) was added to 335 µL of Folin–Ciocalteau reagent and diluted with 1.7 mL of deionised water. After resting in the dark for 3 min, 2.5 mL of 7.5% (*w*/*v*) Na_2_CO_3_ solution was added. The reaction mixture was incubated in the dark for 60 min and then the absorbance was measured at 740 nm against a blank without phenolic extract, using a Cary 50 spectrophotometer (Agilent Technologies). The results were expressed as g gallic acid equivalents (GAE)/100 g dry solid or resins.

#### 2.4.6. Antioxidant Activity

##### Ferric Reducing Antioxidant Power

Ferric reducing antioxidant power (FRAP) was evaluated in the phenolic aqueous extract of spent solid and in the ethanolic solution of soft and hard resins by the method described by Chiancone et al. [23], with some modifications. Briefly, the FRAP reagent solution was prepared by mixing 100 mL of sodium acetate buffer solution (pH 3.6), 10 mL of 10 mmol/L 2,4,6-tris(2-pyridyl)-s-triazine (TPTZ) solution in 40 mmol/L of HCl and 10 mL of 20 mmol/L of FeCl3·6H2O. Then, 100 µL of phenolic extract or ascorbic acid standard solution was mixed with 3.0 mL of FRAP reagent. The resulting solution was pre-incubated in the dark at room temperature for 5 min. Next, the absorbance of the standards and samples was measured at 593 nm, using a Cary 50 spectrophotometer (Agilent Technologies) against a blank containing 100 µL of deionised water. The results were expressed as g ascorbic acid equivalents (AAE)/100 g dry solid or resins.

##### DPPH Radical-Scavenging Activity

The antiradical capacity of the aqueous spent solid extract and ethanolic resins solutions was determined using a 2,2-diphenyl-1-picrylhydrazyl (DPPH) assay according to Squillaci et al. [24], with some modifications. In brief, different concentrations of the extract or resins solution, ranging from 1.5 to 90 µg GAE/mL, were prepared by dilution with water. Then, a volume of 300 µL of the diluted sample was mixed with 2.7 mL of methanolic solution containing 60 µmol/L of DPPH radicals. The mixture was shaken vigorously and left to stand in the dark for 30 min. DPPH radical reduction was then measured by reading the absorbance at 517 nm against a control solution containing 300 µL of deionised water. The radical-scavenging activity (RSA) was calculated as RSA (%) = 100 × [(A0 − AS)/A0], where A0 is the absorbance of the control solution and AS is the absorbance of the diluted sample. The extract concentrations providing maximum radical-scavenging activity (C RSAmax) and 50% of radical-scavenging activity (EC50) were calculated from the plot of RSA (%) vs. extract and resins concentration. The results were expressed as mg sample (spent solid or resins)/mL required to obtain C RSAmax and EC50. Catechin and ascorbic acid were used as standards.

### 2.5. Statistical Analysis

All the samples (except those used to determine the essential oils compounds profile and those of the RSM experimental design) were analysed in triplicate, and data in tables represent mean values ± standard deviation (*n* = 3). Results were assessed for statistical significance using one-way ANOVA and Tukey’s HSD test as a post hoc test using Statistix 10.0 software (Analytical Software, Tallahassee, FL, USA). Differences were considered significant when *p* < 0.05. For the optimisation step, an experimental design based on RSM was planned and analysed using Minitab 16 software (Minitab, State Collage, PA, USA).

## 3. Results and Discussion

### 3.1. Chemical Composition of Six Hop Varieties

As can be seen in Table 1, the levels of bitter acids, xanthohumol, TPC, essential oils and their compounds profile varied according to the type of cultivar [6,14]. Thus, the highest levels of α- and β-acids (*p* < 0.05) were obtained from the Columbus (12.84 ± 0.13 and 4.43 ± 0.02%, respectively) followed by the Nugget cultivars (11.39 ± 0.12 and 4.05 ± 0.04%, respectively), while the lowest levels were found in the Fuggle cultivar (5.20 ± 0.00 and 2.55 ± 0.01%, respectively). In the case of α-acids, the n+adhumulone concentration was at least two times higher than the cohumulone content for all hop varieties. For β-acids, the lupulone content was very similar to the n+adlupulone content for the six hop varieties. These results were in agreement with Chadwick et al. [25] who reported bitter acids in hops in the range of 5–20% (*w*/*w*).

In accordance with other studies [26], all varieties had a xanthohumol content of less than 1% *w*/*w*. Thereby, Nugget and Columbus were the cultivars with the highest levels of xanthohumol (*p* < 0.05) (0.75 ± 0.01%), while Fuggle was the cultivar with the lowest level (0.35 ± 0.00%).

For TPC (excluding xanthohumol), the cultivars with the highest levels (*p* < 0.05) were Cascade (3.60 ± 0.01 g GAE/100 g) followed by Fuggle (3.28 ± 0.03 g GAE/100 g). These results were lower than the polyphenol content described for dried hop cones by Proestos and Komaitis [27] (4–14%, *w*/*w*), although the hop variety was not specified in that work. Furthermore, it is important to mention that the content of TPC depends on the time of harvest [28].

Regarding essential oil content, Columbus and Nugget had the highest levels (*p* < 0.05) (1.70 ± 0.07 and 1.60 ± 0.07% *v*/*w*, respectively), while Cascade and Fuggle had the lowest concentrations (0.75 ± 0.07 and 0.90 ± 0.07% *v*/*w*, respectively). In the case of the essential oil compounds profile, nine compounds were determined and great differences in their contents were observed as a function of the hop cultivar (Table 1). Thus, the acyclic monoterpene myrcene was the main compound in all varieties, with concentrations ranging from 29.70% (relative area, rel) in Chinook to 64.00% (rel) in Magnum. These levels were in agreement with Almaguer et al. [6], who indicated that myrcene may account for 30–60% (rel). Moreover, as cited by other authors [6], the sesquiterpenes α-humulene, β-cariophyllene and β-farnesene were the second most abundant group of compounds. Thus, α-humulene ranged from 14.24% (rel) in Magnum to 25.74% (rel) in Fuggle, β-caryophyllene from 5.96% (rel) in Magnum to 10.39% (rel) in Chinook, and β-farnesene was only detected in Fuggel (4.37%, rel) and Cascade (7.96%, rel) varieties. Concentrations of other quantified compounds, β-pinene and limonene (monoterpenes), linalool and geraniol (terpene alcohols) and 2-undecanone (ketone) were less than 1% (rel) in all hop varieties except for geraniol in Cascade (1.64%, rel) and linalool in Nugget (1.15%, rel). It should be noted that linalool and geraniol are hydration products of myrcene, the major compound of essential oils from hops [29]. The GC chromatograms of the essential oils obtained from the six hop varieties are shown in Figure S1.

### 3.2. Development of a Sequential Extraction and Separation Procedure for Hops

A sequential extraction procedure was developed to recover the main high-value compounds from hops separated into different streams. The aim was to maximise the recovery of α- and β-acids in soft resins, xanthohumol in hard resins and TPC in spent solids. Several methods have been developed for resins extraction from hops, but all have shown the difficulty of achieving a high purification of the resins [6]. Some works have prepared soft and hard resins from hops after an initial extraction using methanol–diethyl ether mixture, followed by other steps including the addiction of hexane to fractionate the total resins [30,31]. However, no studies were found that focused on optimising the extraction and separation of α- and β-acids, xanthohumol and the rest of the phenolic compounds from hops within different streams.

Therefore, a preliminary study was carried out to select the most suitable solvent (methanol, ethanol, dichloromethane, acetone, ethyl acetate, hexane, methanol–diethyl ether mixture 25:75 (*v*/*v*) and 50:50 (*v*/*v*) and methanol–dichloromethane mixture 25:75 (*v*/*v*) and 50:50 (*v*/*v*)) to maximise the extraction of biocompounds from hops pellets (Nugget variety), followed by the fractionation steps described in Section 2.2. The further separation of the hop extract was based on fast and simple methods such as filtration, rotatory evaporation and liquid–liquid (L-L) extraction. The influence of the type of solvent used to extract the biocompounds from hop cones on the subsequent separation efficiency was also investigated. The solvent should be able to extract the maximum amount of α-acids, β-acids and xanthohumol (hydrophobic compounds) from hops and leave the phenolic compounds (hydrophilic compounds) in the spent biomass. In addition, the solvent selected should also maximise the amount of α- and β-acids extracted with hexane in the subsequent fractionation step (separation of soft resins and hard resins), minimising the amount of xanthohumol extracted with hexane, leaving it in hard resins. As can be seen in Table 2, the highest recoveries of α- and β-acids in soft resins (*p* < 0.05) were obtained with dichloromethane (91.55 ± 1.94% and 88.68 ± 1.63%, respectively) followed by methanol–dichloromethane 25:75 (82.16 ± 1.82% and 88.18 ± 1.06%, respectively), ethyl acetate (80.09 ± 0.49% and 86.15 ± 1.98%, respectively) and hexane (78.95 ± 1.25% and 85.59 ± 1.92%, respectively). Several studies have reported successful results in the extraction of bitter acids from hop cones using dichloromethane, methanol and hexane [5,26]. In relation to the results obtained in this work, although ethyl acetate led to a high recovery of α- and β-acids in soft resins, this solvent also led to a high recovery of xanthohumol in soft resins, reducing its separation efficiency from bitter acids. Similarly, dichloromethane and hexane were among the solvents that gave the lowest xanthohumol recovery in hard resins. It is important to mention that TPC could not be determined in soft resins because several chemical interferences were observed when applying the Folin–Ciocalteu’s method to this stream.

Regarding hard resins, the recovery of xanthohumol reached the highest values (*p* < 0.05) when the S-L extraction from cone hop pellets was carried out with methanol–dichloromethane 25:75 (78.99 ± 0.81%), methanol (78.55 ± 0.46%) or methanol–dichloromethane 50:50 (77.43 ± 0.42%), followed by hexane fractionation of the total resins (Table 2). It should be noted that methanol and methanol–dichloromethane 50:50 were also among the solvents that led to an increase in the recovery values of α- and β-acids and TPC in hard resins (an effect to avoid). Furthermore, in relation to methanol –dichloromethane and methanol–diethyl ether mixtures, it can be observed that increasing the methanol percentage increases the recovery of bitter acids and TPC in hard resins. Previous studies have focused on conventional methods for obtaining xanthohumol from hops, which consist of several steps, generally involving extraction with organic solvents such as dichloromethane, methanol, chloroform, ethyl acetate, hexane, diethyl ether and acetone, followed by other separation steps such as chromatography, precipitation or crystallisation [32].

Regarding the TPC, the aim was to select a solvent with a low affinity for them (low extraction efficiency) so that they remained concentrated in the spent solids and could be separated from the other hop biocompounds. As can be seen in Table 2, the highest levels of TPC in the spent solids (*p* < 0.05) were obtained when hop pellets were extracted with ethyl acetate (74.57 ± 1.73%) or dichloromethane (72.08 ± 2.51%) followed by hexane (64.74 ± 2.02%), acetone (64.16 ± 2.28) or methanol–dichloromethane 25:75 (64.14 ± 0.42%). These results were in agreement with those obtained by Önder et al. [33] and Veiga et al. [2], who tested several organic solvents for the extraction of TPC from hops and obtained higher extraction yields when ethanol or methanol were used neat or in aqueous solution than those obtained when hexane or ethyl acetate were used as extraction solvents. Furthermore, repeated studies in the literature have used aqueous methanol or ethanol as well as boiling water [5,9,14,34] to extract TPC from hops, with successful results. Thus, with respect to the solvents that presented low extraction efficiency of TPC from hops, dichloromethane, ethyl acetate and hexane led to obtaining hard resins with low values of xanthohumol recovery and acetone led to obtaining soft resins with low values of bitter acids recovery.

Since methanol–dichloromethane mixtures (with a percentage of methanol lower than 50%) allowed the highest recovery yields of the four hops components separated into three streams (soft resins, hard resins and spent solids), this mixture was chosen to optimise the S-L extraction process from hops. Another aspect worth mentioning is that methanol, dichloromethane and hexane are authorised to be used as solvents for the extraction of compounds from natural aromatic plants with regulated maximum residual limits in food products, according to Directive 2009/32/EC [10].

### 3.3. Optimisation of High-Value Compounds Extraction from Hops

In order to maximise the recovery of high-value compounds from hop pellets separated into different streams by sequential extraction, two independent variables of the initial S-L extraction, using methanol–dichloromethane, were optimised by applying RSM with an experimental CCRD. Table 3 summarises the combinations of the two independent variables (methanol concentration and stirring time) and the four observed response values (recovery yields of α-acids and β-acids in soft resins, xanthohumol in hard resins and TPC in spent solids). All the recoveries of high-value compounds from hops determined in the three recovered fractions are shown in Table S1. The thirteen sequential extraction trials yielded variable recoveries ranging from 70.43 to 88.03% (α-acids in soft resins), 79.71 to 92.50% (β-acids in soft resins), 65.47 to 80.28% (xanthohumol in hard resins) and 59.76 to 76.27% (TPC in spent solids).

**Figure 2:**
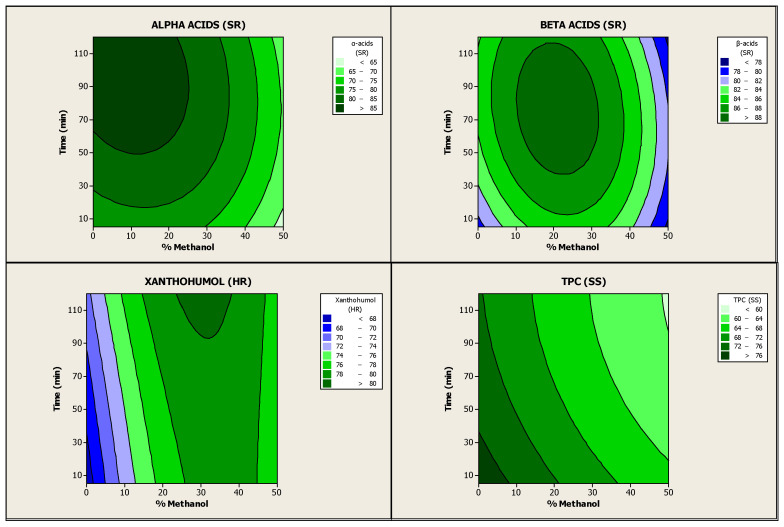
Contour plots between coupled recovery yields of α- and β-acids in soft resins, xanthohumol in hard resins and TPC in spent solids as a function of methanol concentration (%, *v*/*v*) and stirring or extraction time (min).

An ANOVA was then performed to analyse these experimental results and to evaluate the effects of the independent variables and their possible interactions. The calculated quadratic model explained 82.84% of the variation of α-acids in soft resins (Table S2), 51.34% of the variation of β-acids in soft resins (Table S3), 75.85% of the variation of xanthohumol in hard resins (Table S4) and 90.63% of the variation of TPC in spent solids (Table S5), indicating that it can be only moderately accepted. Nevertheless, even though the model was overfitted, the predicted quadratic model for the recoveries of α-acids in soft resins, xanthohumol in hard resins and TPC in spent solids is still significant at 95% confidence level with p-values less than 0.05 (0.013, 0.039 and 0.002, respectively) (Tables S6–S9). Figure 2 shows the contour plots associated with the effect of S-L extraction of hop pellets followed by sequential fractionation on the abovementioned recovery yields. The contour plots confirm that there were significant interactions between methanol concentration and stirring time on the four response variables analysed. In addition, methanol concentration had a more significant influence on the values of these recovery rates than stirring time. Furthermore, the effect of these independent variables was positive for all the response variables, except for TPC in spent solids, where it was negative. In this case, the recovery yield decreased with increasing methanol concentration and stirring time as the TPC extraction increased [35]

The optimum values of methanol concentration and stirring time were calculated using the equations of the model in order to achieve maximum recovery yields of the high-value hop compounds in the abovementioned fractions. The optimisation results indicated that the best parameters for the S-L extraction of hop pellets (Nugget variety) carried out with methanol–dichloromethane as extraction solvent at room temperature and with a fixed value of 5% (*w*/*w*) of hops loading were 19.7% (*v*/*v*) methanol (80.3% *v*/*v* dichloromethane) and 89 min of stirring time. Under these conditions, the estimated recovery yields were: 86.57% α-acids and 89.14% β-acids in soft resins, 78.48% xanthohumol in hard resins and 67.10% TPC in spent solids. The optimal operating conditions were experimentally validated by extraction of hop pellets (Nugget variety) followed by sequential fractionation, in triplicate. After the validation, the recovery results attained were: 81.67 ± 1.52% α-acids in soft resins, 88.00 ± 1.38% β-acids in soft resins, 79.65 ± 0.78% xanthohumol in hard resins and 67.07 ± 1.31% TPC in spent solids (Table 4). Some small differences between calculated and experimental values were observed (especially in the case of α-acids in soft resins), but in general, the results confirmed that the response model was adequate and valid enough to calculate the optimisation results.

**Figure 3:**
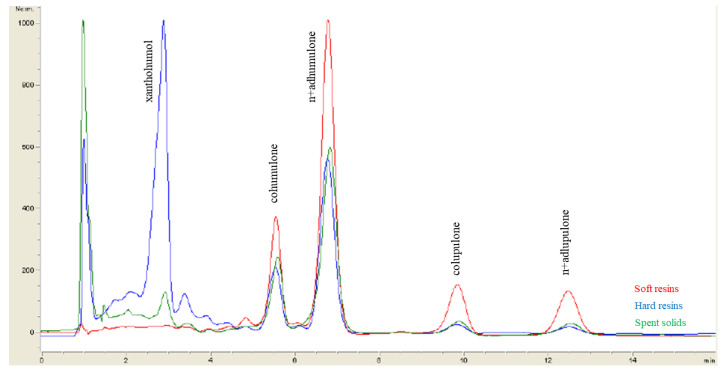
Comparison of the HPLC-DAD chromatograms of the soft resins, the hard resins and the spent solids obtained by applying the optimum conditions for the S-L extraction of hop pellets (Nugget variety) followed by sequential extraction. Note: The α-acids concentration was calculated as the sum of cohumulone and n+adhumulone and the β-acids concentration as the sum of colupulone and n+adlupulone.

Figure 3 shows the HPLC-DAD chromatograms of α-acids, β-acids and xanthohumol in the soft resins, the hard resins and the spent solids obtained from hop pellets (Nugget variety) under optimum operating conditions for the initial S-L extraction followed by fractionation.

Several authors have successfully used the RSM approach to optimise the operating conditions of S-L extraction of biocompounds from biomass. Some examples are the studies carried out by Piechowiak et al. [36], that maximised the extraction yield, TPC recovery and antioxidant activity from yellow onion skin, by Silva et al. [37], that maximised the yield of TPC, total flavonoids and total flavonols extracted from *Inga edulis* and by Vázquez et al. [38], that maximised the yield of TPC from chestnut burr. Futhermore, Lorbeer et al. [11] successfully optimised the acid treatment of the alga *Ecklonia radiata* to extract fucodian and improve the efficient sequential extraction of alginates based on conventional methods. Regarding the optimisation of extraction from hops by RSM, Lakka et al. [39] improved the efficient recovery of TPC using deep eutectic solvents, Nagybákay et al. [40] used supercritical CO_2_ extraction to produce extracts with high yield and strong antioxidant properties and Almeida et al. [41] obtained the most effective extraction conditions to maximise TPC recovery from Brazilian hops. Nevertheless, no studies were found in the literature that focused on optimising the recovery and fractionation of the main high-value compounds of hops (bitter acids, xanthohumol and phenolic compounds), separated into different streams, in order to simultaneously maximise their recovery yields. 

It should be noted that the main drawback of the sequential extraction process developed in this work is the need for distillation steps to remove the organic solvents from the extract. Although the distilled solvent could be re-used in the process, its evaporation could remove volatile compounds (especially essential oils) from the extract and cause degradation of thermolabile compounds. To overcome these problems, the use of techniques such as sc-CO_2_ can provide purer extracts but can only be used to recover apolar fractions such as bitter acids and essential oils. However, to extract and fractionate the high-value polar compounds of hops (xanthohumol and phenolic compounds) the sc-CO_2_ technique also requires the use of a co-solvent (organic solvent) to be effective [2].

### 3.4. Application of the Optimal S-L Extraction Conditions to Other Hop Varieties: Chemical Composition of the Hop-Derived Fractions

In addition to the Nugget variety, the optimum values calculated for the experimental variables of the S-L extraction were applied to five bitter and aromatic hop varieties (pellet form): Cascade, Columbus, Fuggle, Magnum and Chinook, with the subsequent sequential fractionation steps. The high-value compounds in the streams obtained were then analysed in order to calculate their recovery yields (Table 4). Thus, the highest recoveries of α-acids in soft resins (*p* < 0.05) were achieved with Magnum (84.89 ± 2.57%) and Nugget (81.67 ± 1.52%) cultivars. For β-acids in soft resins, the maximum values were reached with Magnum (89.87 ± 2.93%), Cascade (88.06 ± 1.84%), Nugget (88.00 ± 1.38%), Fuggle (87.97 ± 2.20%) and Chinook (83.82 ± 2.79%) varieties, with no significant differences between them (*p* < 0.05). Regarding xanthohumol in hard resins, the highest recovery (*p* < 0.05) was attained with Nugget (79.65 ± 0.78%) and the lowest with Columbus (66.00 ± 1.36%). For TPC in spent solids, the highest yield (*p* < 0.05) was obtained with Fuggle (86.62 ± 2.86%) and the lowest with Nugget (67.07 ± 1.04%).

The concentrations (g/100 g) of α- and β-acids, xanthohumol and TPC in initial hops, soft resins, hard resins and spent solids obtained from the six hop varieties under optimal S-L extraction conditions followed by fractionation steps are shown in Table S10. Regarding the soft resins, the main compounds were α-acids (from 26.54 ± 0.38 g/100 g in Cascade to 43.22 ± 2.84 g/100 g in Chinook) and β-acids (from 13.31 ± 0.54 g/100 g in Chinook to 29.29 ± 0.78 g/100 g in Cascade). In the hard resins, the metabolites with the highest concentration were xanthohumol (from 4.28 ± 0.88 g/100 g in Cascade to 9.13 ± 0.74 g/100 g in Magnum) and α-acids (ranging from 5.54 ± 0.93 g/100 g in Chinook to 12.39 ± 1.21 in Columbus), especially ad+humulone (ranging from 3.85 ± 0.70 g/100 g in Chinook to 8.86 ± 0.96 g/100 g in Columbus). The observed concentrations of α- and β-acids in soft resins and xanthohumol in hard resins were comparable to those reported by Kontek et al. [13]. In that work, an extract of hops (Polish cultivar Marynka) was prepared with 80% methanol by sonication and subjected to fractionation and purification by L-L extraction and preparative liquid chromatography, and then was separated into soft resins and hard resins. Nevertheless, Kontek et al. [13] reported higher levels of α-acids in hard resins (27.45 g/100 g) and lower values of concentration of β-acids in hard resins (below the lower limit of quantification), indicating a lower efficiency for the separation of α-acids and a higher efficiency for β-acids than achieved in the present work.

Regarding the spent solids, TPC (excluding xanthohumol) were the major high-value compounds, ranging from 1.75 ± 0.04 g GAE/100 g (Columbus) to 3.56 ± 0.17 g/100 g GAE (Fuggle). These results were higher than those reported by Kowalczyk et al. [34], who extracted phenolics from hops (Magnum variety) and their pellets using water in a shaking bath at 40 °C for 1 h and obtained recoveries ranging from 1.62 to 1.81 g GAE/100 g. However, in that study, higher recoveries were obtained when the solvent extraction was 50% ethanol (4.42–7.35 g GAE/100 g) or 50% methanol (3.87–5.38 g GAE/100 g). Nevertheless, the TPC values obtained from the spent solids in the present work were comparable to those reported by Arruda et al. [42], who used ten hop varieties to obtain ethanolic extracts by maceration at 60 °C for 24 h. Furthermore, Labieniec-Watala et al. [43] performed an extraction of the TPC contained in spent hops (previously subjected to sc-CO_2_ extraction) with a mixture of acetone and water, and the TPC content in the dry extract was determined. However, the cited work did not mention the TPC content in the spent hops, nor the extraction yield, so those contents could not be compared with the results obtained in the present work.

### 3.5. Antioxidant Activities of Hop-Derived Fractions

The antioxidant activities of the three fractions (soft resins, hard resins and spent solids) derived from the six hop varieties were measured using two assays: FRAP and DPPH radical scavenging (RSA). 

Regarding the FRAP results (Table 5), the maximum values were achieved with soft resins and varied from 3.16 ± 0.11 g AAE/100 g (Chinook) to 3.91 ± 0.10 g AAE/100 g (Cascade), without significant differences (*p* < 0.05) among the six varieties. For hard resins, FRAP values ranged from 1.45 ± 0.38 g AAE/100 g (Magnum) to 2.35 ± 0.09 g AAE/100 g (Fuggle) and, for spent solids, from 1.16 ± 0.07 g AAE/100 g (Nugget) to 2.75 ± 0.05 g AAE/100 g (Fuggle).

**Figure 4:**
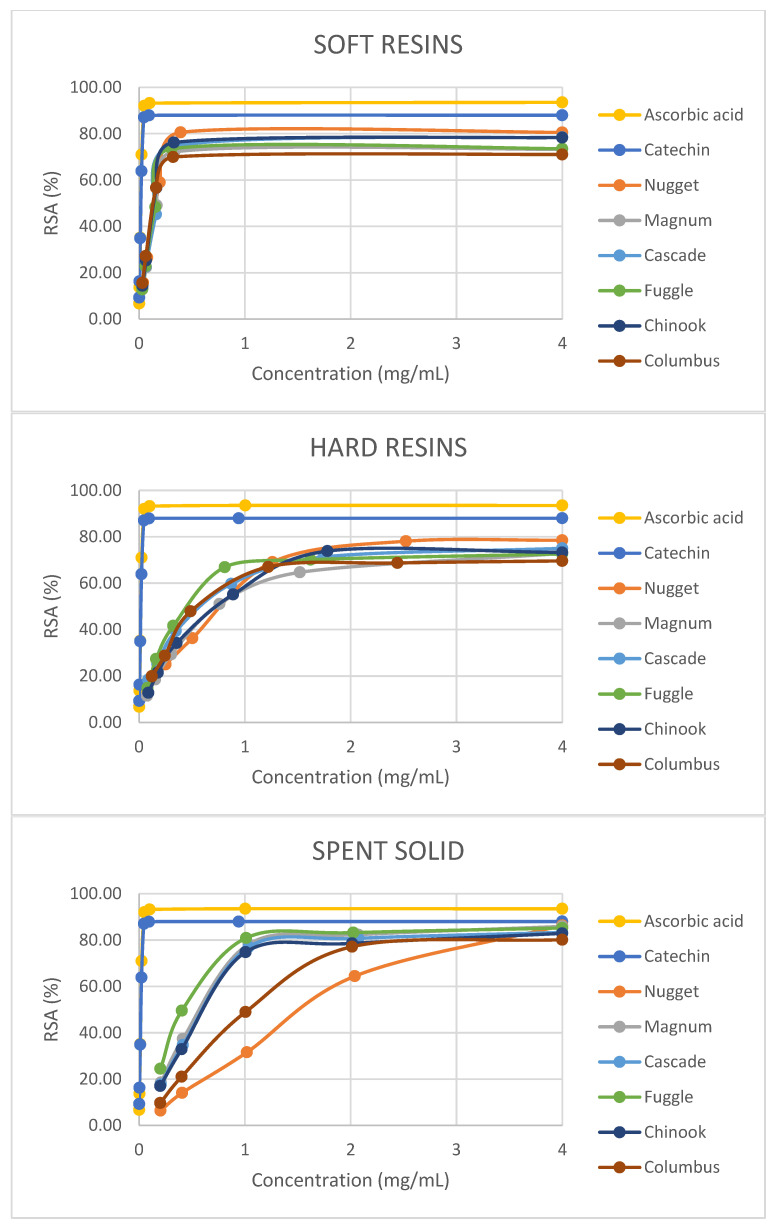
DPPH free-radical-scavenging activity (RSA) of soft resins, hard resins and spent solids concentrations obtained from six hop varieties (pellet form) by sequential fractionation under optimal conditions of S-L extraction and antioxidant reference compounds (ascorbic acid and catechin).

With regard to the results obtained with the DPPH radical-scavenging assay, the RSA values were expressed as the ratio percentage between the decrease in absorbance of the sample and the absorbance of the DPPH solution in the absence of the hop fraction at 517 nm. In Figure 4, it can be seen that the scavenging activity of all the hop-derived fractions on DPPH radicals increased with increasing concentration and reached values close to those attained by ascorbic acid or catechin (reference antioxidant compounds). Furthermore, the highest RSA_max_ values (*p* < 0.05) were achieved with spent solids, ranging from 74.82% at 1.01 mg/mL (Chinook) to 86.36% at 5.10 mg/mL (Nugget) (Table 5). In the case of soft resins, with high humulones and lupulones content, RSA_max_ values varied from 69.93% at 0.32 mg/mL (Columbus) to 80.57% at 0.39 mg/mL (Nugget) and these values were in agreement with those reported by Ting et al. [44] for humulone (74% of RSA_max_) and colupulone (77% of RSA_max_). For hard resins, RSA_max_ values varied from 67.00% at 1.22 mg/mL (Columbus) to 78.08% at 2.52 mg/mL (Nugget). On the other hand, the best EC50 results (*p* < 0.05) were attained with soft resins, with values ranging from 0.14 ± 0.01 mg/mL (Columbus and Chinook) to 0.21 ± 0.00 mg/mL (Magnum). For hard resins, EC50 values were between 0.51 ± 0.02 mg/mL (Fuggle) and 0.83 ± 0.02 mg/mL (Nugget) and for spent solids between 0.51 ± 0.00 mg/mL (Fuggle) to 1.59 ± 0.02 mg/mL (Nugget). Regarding EC50 values for hard resins, the results obtained were comparable to those reported by Kontek et al. [13] for Marynka hop (0.484 ± 0.003 mg/mL), while the EC50 values for soft resins were lower than those mentioned in the cited paper (0.294 ± 0.002 mg/mL). Besides, other works [33,45] reported a stronger activity against DPPH for hop extracts rich in bitter acids than those rich in prenylflavonoids.

It is important to mention that the fractions derived from Fuggle hop achieved higher FRAP and lower EC50 values (*p* < 0.05) than those attained from the abovementioned five hop varieties. Nevertheless, the fractions obtained from Nugget achieved higher RSA_max_ values (*p* < 0.05). 

It can be concluded that, for all hop varieties, the soft resins showed better antioxidant properties than the other two fractions recovered from the hop pellets (hard resins and spent solids). However, it should be noted that the content of bitter acids in the soft resins is rather high (α-acids ranging from 26.54 ± 0.38 to 43.22 ± 2.84 g/100 g and β-acids from 13.31 ± 0.54 to 29.29 ± 0.78 g/100 g), whereas the contents of xanthohumol and phenolic compounds in the corresponding enriched fractions were much lower (ranging from 4.28 ± 0.88 to 9.13 ± 0.74 g/100 g for xanthohumol in hard resins and from 1.75 ± 0.04 to 3.56 ± 0.17 g/100 g for phenolic compounds in spent solids). Therefore, it cannot be concluded that bitter acids have higher antioxidant properties than xanthohumol or phenolic compounds in hops.

## 4. Conclusions

A sequential extraction method to recover the high-value compounds from hops, separated into different fractions, has been developed. The variables of the initial S-L extraction were optimised in order to recover the maximum levels of α- and β-acids in soft resins, xanthohumol in hard resins and TPC in spent solids. The use of 19.7% (*v*/*v*) methanol in dichloromethane for solvent extraction and a stirring time of 89 min at room temperature maximised the recovery of the abovementioned compounds by increasing the extraction of bitter acids and xanthohumol, while decreasing TPC, facilitating their separation in different streams. The application of the optimised extraction conditions to six hop varieties, followed by fractionation steps, produced streams with high concentrations of the separated compounds as well as antioxidant activities. Moreover, the antioxidant activities of the soft resins obtained by the optimised procedure were higher than those of the hard resins and spent solids (with differences between varieties), and comparable to those of the reference antioxidant. These results suggest that the optimised sequential extraction approach could be the first step in a forthcoming industrial scale-up process. However, further research is needed to develop and optimise new cleaner and more sustainable technologies, such as microwave-assisted extraction or ultrasound-assisted extraction, so that they can be easily implemented in industry.

## Figures and Tables

**Table 1:** Chemical composition (dry basis): α-acids (cohumulone, n+adhumulone), β-acids (colupulone, n+adlupulone), xanthohumol, total phenolic compounds (TPC), essential oils content and essential oil compounds profile (%, rel) of six hop varieties. Within chemical composition, for each parameter within each file, different letters mean significant differences (Tukey’s HSD; *p* < 0.05).

Hop Variety	Nugget	Columbus	Chinook	Magnum	Cascade	Fuggle
Chemical compostion (dry basis)						
Cohumulone (%, *w*/*w*)	2.53 ± 0.04 _C_	3.77 ± 0.05 _A_	3.10 ± 0.12 _B_	2.23 ± 0.01 _D_	1.75 ± 0.01 _E_	1.75 ± 0.06 _E_
n+Adhumulone (%, *w*/*w*)	8.86 ± 0.08 _A_	9.07 ± 0.08 _A_	6.88 ± 0.18 _B_	5.39 ± 0.07 _C_	3.39 ± 0.01 _D_	5.17 ± 0.04 _C_
Lupulone (%, *w*/*w*)	2.02 ± 0.02 _B_	2.46 ± 0.00 _A_	1.61 ± 0.06 _CD_	1.65 ± 0.02 _C_	2.53 ± 0.02 _A_	1.54 ± 0.02 _D_
n+Adlupulone (%, *w*/*w*)	2.03 ± 0.02 _B_	1.97 ± 0.02 _B_	1.25 ± 0.03 _D_	1.27 ± 0.02 _D_	2.50 ± 0.03 _A_	1.75 ± 0.02 _C_
α-Acids (%, *w*/*w*)	11.39 ± 0.12 _B_	12.84 ± 0.13 _A_	9.97 ± 0.00 _C_	7.61 ± 0.06‘_D_	6.92 ± 0.01 _E_	5.20 ± 0.00 _F_
β-Acids (%, *w*/*w*)	4.05 ± 0.04 _B_	4.43 ± 0.02 _A_	2.85 ± 0.02 _D_	2.92 ± 0.01 _D_	3.28 ± 0.04 _C_	2.55 ± 0.01 _E_
Xanthohumol (%, *w*/*w*)	0.76 ± 0.01 _A_	0.75 ± 0.01 _A_	0.61 ± 0.02 _B_	0.59 ± 0.00 _B_	0.44 ± 0.00 _C_	0.35 ± 0.00 _D_
TPC (g GAE/100 g)	2.33 ± 0.02 _E_	1.88 ± 0.03 _F_	3.04 ± 0.03 _D_	3.13 ± 0.02 _C_	3.60 ± 0.01 _A_	3.28 ± 0.03 _B_
Essential oils (%, *v*/*w*)	1.60 ± 0.07 _A_	1.70 ± 0.07 _A_	1.20 ± 0.07 _B_	1.20 ± 0.07 _B_	0.75 ± 0.07 _C_	0.90 ± 0.07 _C_
Essential oils composition						
β-Pinene (%, rel)	0.16	0.66	0.23	0.37	0.40	0.25
Myrcene (%, rel)	51.84	56.43	29.70	64.00	44.18	42.30
Limonene (%, rel)	0.66	0.66	0.50	0.84	0.61	0.61
Linalool (%, rel)	1.15	0.77	0.59	0.63	0.61	0.75
Geraniol (%, rel)	0.08	0.75	0.51	0.62	1.64	0.27
2-Undecanone (%, rel)	0.52	0.19	0.32	0.62	0,24	0.49
β-Cariophyllene (%, rel)	9.01	7.69	10.39	5.96	7.34	9.75
β-Farnesene (%, rel)	nd	nd	nd	nd	7.96	4.37
Humulene (%, rel)	19.84	15.03	24.42	14.29	18.16	25.74

GAE: Gallic acid equivalents.

**Table 2:** Recovery yields (%) of the valuable hop compounds in soft resins, hard resins and spent solids obtained using different solvents for S-L extraction followed by fractionation steps. Within each fraction for each column, different letters mean significant differences (Tukey’s HSD; *p* < 0.05).

Solvent	α-Acids	β-Acids	Xanthohumol	TPC
	(Yield, %)	(Yield, %)	(Yield, %)	(Yield, %)
Soft resins				
Methanol	54.28 ± 0.57 _F_	74.77 ± 1.33 _F_	5.93 ± 0.69 _CD_	na
Ethanol	69.69 ± 0.79 _DE_	80.54 ± 0.71 _CDE_	11.61 ± 2.70 _AB_	na
Dichloromethane	91.55 ± 1.94 _A_	88.68 ± 1.63 _A_	10.05 ± 1.12 _BC_	na
Acetone	57.67 ± 1.02 _F_	78.45 ± 1.25 _DEF_	5.76 ± 0.70 _CD_	na
Ethyl Acetate	80.09 ± 0.49 _BC_	86.15 ± 1.98 _AB_	14.83 ± 1.01 _A_	na
Hexane	78.95 ± 1.25 _BC_	85.59 ± 1.92 _AB_	4.12 ± 0.47 _D_	na
Methanol–Diethyl Ether (25:75)	77.01 ± 1.24 _C_	83.23 ± 1.44 _BC_	9.03 ± 0.73 _BC_	na
Methanol–Diethyl Ether (50:50)	65.73 ± 1.85 _E_	76.43 ± 1.29 _EF_	10.49 ± 2.54 _AB_	na
Methanol–Dichloromethane (25:75)	82.16 ± 1.82 _B_	88.18 ± 1.06 _A_	8.36 ± 2.05 _BCD_	na
Methanol–Dichloromethane (50:50)	70.03 ± 1.99 _D_	80.93 ± 1.44 _CD_	9.24 ± 0.93 _BC_	na
Hard resins				
Methanol	30.10 ± 1.52 _A_	13.00 ± 2.05 _A_	78.55 ± 0.46 _AB_	61.98 ± 2.62_A_
Ethanol	14.84 ± 0.45 _CD_	4.77 ± 0.52 _CD_	74.38 ± 1.38 _CD_	14.15 ± 0.86 _CD_
Dichloromethane	1.50 ± 0.18 _G_	0.78 ± 0.13 _E_	64.64 ± 1.48 _F_	1.77 ± 0.81 _E_
Acetone	27.19 ± 0.77 _A_	10.43 ± 1.22 _AB_	73.62 ± 0.36 _DE_	17.65 ± 1.32 _C_
Ethyl Acetate	5.83 ± 0.25 _F_	1.26 ± 0.07 _E_	64.76 ± 1.21 _F_	3.79 ± 0.30 _E_
Hexane	12.36 ± 0.55 _DE_	2.87 ± 0.18 _DE_	64.89 ± 1.97 _F_	15.44 ± 1.07 _CD_
Methanol–Diethyl Ether (25:75)	10.43 ± 1.38 _E_	3.89 ± 0.71 _DE_	70.92 ± 1.22 _E_	11.65 ± 0.89 _D_
Methanol–Diethyl Ether (50:50)	22.84 ± 2.14 _B_	11.22 ± 1.66 _A_	75.29 ± 1.11 _BCD_	23.56 ± 2.44 _B_
Methanol–Dichloromethane (25:75)	9.69 ± 2.24 _E_	2.82 ± 1.69 _DE_	78.99 ± 0.81 _A_	14.56 ± 2.59 _CD_
Methanol–Dichloromethane (50:50)	18.08 ± 0.89 _C_	7.73 ± 1.04 _BC_	77.43 ± 0.42 _ABC_	24.93 ± 0.52 _B_
Spent solids				
Methanol	3.09 ± 0.89 _BCD_	1.92 ± 0.74 _BC_	2.67 ± 0.87 _D_	39.19 ± 2.03 _D_
Ethanol	4.74 ± 1.08 _AB_	2.89 ± 0.95 _AB_	5.26 ± 1.22 _C_	58.86 ± 1.14 _C_
Dichloromethane	2.21 ± 0.17 _CDE_	0.35 ± 0.27 _CD_	15.27 ± 0.70 _A_	72.08 ± 2.51 _A_
Acetone	3.67 ± 0.44 _BC_	1.66 ± 0.36 _BCD_	8.77 ± 0.41 _B_	64.16 ± 2.28 _B_
Ethyl Acetate	5.81 ± 1.23 _A_	3.65 ± 1.33 _A_	15.01 ± 1.14 _A_	74.57 ± 1.73 _A_
Hexane	1.08 ± 0.08 _E_	0.00 ± 0.00 _D_	6.92 ± 0.58 _BC_	64.74 ± 2.02 _B_
Methanol–Diethyl Ether (25:75)	2.25 ± 0.30 _CDE_	1.12 ± 0.24 _CD_	7.78 ± 0.37 _B_	58.96 ± 1.28 _C_
Methanol–Diethyl Ether (50:50)	1.57 ± 0.29 _DE_	0.70 ± 0.20 _CD_	2.82 ± 0.46 _D_	57.76 ± 1.19 _C_
Methanol–Dichloromethane (25:75)	1.50 ± 0.06 _DE_	0.43 ± 0.06 _CD_	1.91 ± 0.18 _D_	64.14 ± 0.42 _B_
Methanol–Dichloromethane (50:50)	1.47 ± 0.08 _DE_	0.47 ± 0.05 _CD_	1.33 ± 0.09 _D_	54.76 ± 1.30 _C_

TPC: Total phenolic compounds. na: Not analysed.

**Table 3:** Experimental design and the observed results of recovery yields (%) according to the Central Composite Rotatable Design.

Trial	Methanol Concentration (% *v*/*v*)	Stirring Time (min)	α-Acids in Soft Resins Yield (%)	β-Acids in Soft Resins Yield (%)	Xanthohumol in Hard Resins Yield (%)	TPC in Spent Solids Yield (%)
1	25	62.5	88.03	92.50	80.28	65.86
2	25	62.5	78.59	82.66	78.48	67.76
3	50	62.5	70.43	82.45	78.01	61.89
4	25	62.5	81.23	86.06	79.20	65.42
5	25	62.5	86.74	90.91	79.96	65.52
6	25	120	87.99	91.50	78.69	66.45
7	7	21.8	79.81	82.24	74.43	76.27
8	25	5.0	75.96	86.46	77.07	69.47
9	43	103.2	71.90	79.71	78.65	59.76
10	0	62.5	87.45	87.16	65.47	73.58
11	25	62.5	81.89	85.61	77.05	69.22
12	43	21.8	71.31	81.29	77.56	65.77
13	7	103.2	83.91	83.00	77.71	69.83

TPC: Total phenolic compounds.

**Table 4:** Predicted responses (calculated by the mathematical model for the hop variety Nugget) and observed responses under optimal conditions for the two-independent model. For each file, different letters mean significant differences (Tukey’s HSD; *p* < 0.05).

	Calculated	Observed					
	(Yield, %)	(Yield, %)					
	Nugget	Nugget	Columbus	Chinook	Magnum	Cascade	Fuggle
α-Acids in soft resins	86.57	81.67 ± 1.52 _AB_	62.84 ± 1.67 _C_	77.92 ± 1.08 _B_	84.89 ± 2.57 _A_	78.21 ± 1.21 _B_	78.76 ± 1.28 _B_
β-Acids in soft resins	89.14	88.00 ± 1.38 _A_	72.58 ± 2.18 _B_	83.82 ± 2.79 _A_	89.87 ± 2.93 _A_	88.06 ± 1.84 _A_	87.97 ± 2.20 _A_
Xanthohumol in hard resins	78.48	79.65 ± 0.78 _A_	66.00 ± 1.36 _C_	72.76 ± 0.61 _B_	72.27 ± 0.94 _B_	70.06 ± 0.65 _B_	72.28 ± 2.13 _B_
TPC in spent solids	67.10	67.07 ± 1.04 _D_	72.86 ± 1.87 _C_	79.14 ± 2.26 _B_	77.72 ± 2.22 _BC_	78.42 ± 1.40 _BC_	86.62 ± 2.86 _A_

TPC: Total phenolic compounds.

**Table 5:** Antioxidant activities of fractions recovered from six varieties of hops under optimal S-L extraction conditions following by sequential fractionation. Within each fraction for each file, different letters mean significant differences (Tukey’s HSD; *p* < 0.05).

	Nugget	Columbus	Chinook	Magnum	Cascade	Fuggle
Soft resins						
FRAP (g AAE/100 g)	3.46 ± 0.05 _A_	3.43 ± 0.09 _A_	3.16 ± 0.11 _A_	3.26 ± 0.17 _A_	3.91 ± 0.10 _A_	3.84 ± 0.23 _A_
(*) RSA_max_ (%)	80.57 ± 0.21 _A_	69.93 ± 0.17 _F_	76.18 ± 0.32 _B_	72.16 ± 0.22 _E_	74.63 ± 0.17 _C_	73.50 ± 0.33 _D_
(*) C RSA_max_ (mg/mL)	0.39 ± 0.02 _A_	0.32 ± 0.02 _B_	0.33 ± 0.03 _B_	0.34 ± 0.01 _AB_	0.32 ± 0.01 _B_	0.31 ± 0.01 _B_
(*) EC50 (mg/mL)	0.16 ± 0.01 _B_	0.14 ± 0.00 _C_	0.14 ± 0.01 _C_	0.21 ± 0.00 _A_	0.20 ± 0.00 _A_	0.16 ± 0.00 _B_
Hard resins						
FRAP (g AAE/100 g)	1.84 ± 0.01 _AB_	1.89 ± 0.09 _AB_	2.05 ± 0.30 _AB_	1.45 ± 0.38 _B_	1.86 ± 0.16 _AB_	2.35 ± 0.09 _A_
(*) RSA_max_ (%)	78.08 ± 0.17 _A_	67.00 ± 0.15 _F_	73.82 ± 0.30 _B_	72.83 ± 0.32 _C_	71.28 ± 0.22 _D_	69.90 ± 0.23 _E_
(*) C RSA_max_ (mg/mL)	2.52 ± 0.03 _B_	1.22 ± 0.02 _D_	1.78 ± 0.03 _C_	3.80 ± 0.05_A_	1.74 ± 0.02 _C_	0.81 ± 0.02 _E_
(*) EC50 (mg/mL)	0.83 ± 0.02 _A_	0.74 ± 0.01 _B_	0.76 ± 0.02 _B_	0.72 ± 0.02 _B_	0.65 ± 0.01 _C_	0.51 ± 0.02 _D_
Spent solids						
FRAP (g AAE/100 g)	1.16 ± 0.07 _C_	1.29 ± 0.02 _C_	2.15 ± 0.05 _B_	2.17 ± 0.03 _B_	2.06 ± 0.01 _B_	2.75 ± 0.05 _A_
(*) RSA_max_ (%)	86.36 ± 0.30 _A_	77.15 ± 0.21 _D_	74.82 ± 0.35 _E_	82.31 ± 0.21 _B_	80.76 ± 0.18 _C_	80.91 ± 0.32 _C_
(*) C RSA_max_ (mg/mL)	5.10 ± 0.07 _A_	2.01 ± 0.01 _B_	1.01 ± 0.02 _C_	2.07 ± 0.03 _B_	2.07 ± 0.02 _B_	1.01 ± 0.01 _C_
(*) EC50 (mg/mL)	1.59 ± 0.02 _A_	1.20 ± 0.00 _B_	0.66 ± 0.01 _C_	0.63 ± 0.01 _D_	0.65 ± 0.01 _CD_	0.51 ± 0.00 _E_

FRAP: Ferric reducing antioxidant power. (*): 2,2-diphenyl-1-picrylhydrazyl (DPPH) radical-scavenging assay. RSAmax: Maximum DPPH radical-scavenging activity. C RSAmax: Concentration of a substance that provides maximum DPPH radical-scavenging activity. EC50: Concentration of a substance that provides 50% of DPPH radical-scavenging activity. AAE: Ascorbic acid equivalents. Ascorbic acid (RSAmax: 92.0 ± 0.2%; C RSAmax: 0.05 ± 0.00 mg/mL; EC50: 0.02 ± 0.00 mg/mL). Catechin (RSAmax: 87.1 ± 0.2%; C RSAmax: 0.05 ± 0.00 mg/mL; EC50: 0.02 ± 0.00 mg/mL).

## Data Availability

All data generated or analysed during this study are included in this published article and its Supplementary Materials files.

## Supplementary Materials

The following supporting information can be downloaded at: https://www.mdpi.com/article/10.3390/antiox13010045/s1, Figure S1: GC chromatograms of the essential oils obtained from the six hop varieties (Nugget, Columbus, Chinook, Magnum, Cascade and Fuggle); Table S1: Experimental results of the recoveries (%) of α- and β-acids, xanthohumol and total phenolic compounds (TPC) in soft resins, hard resins and spent solids according to experimental design; Table S2: Estimated regression coefficients for the recovery of α-acids in soft resins; Table S3: Estimated regression coefficients for the recovery of β-acids in soft resins; Table S4: Estimated regression coefficients for the recovery of xanthohumol in hard resins; Table S5: Estimated regression coefficients for the recovery of total phenolic compounds (TPC) in spent solids; Table S6: Analysis of variance for the recovery of α-acids in soft resins; Table S7: Analysis of variance for the recovery of β-acids in soft resins; Table S8: Analysis of variance for the recovery of xanthohumol in hard resins; Table S9: Analysis of variance for the recovery of total phenolic compounds (TPC) in spent solids; Table S10: Weight and chemical composition (α- and β-acids, xanthohumol and total phenolic compounds (TPC)) of the initial hop pellets and the fractions obtained after the sequential extraction process carried out under optimal S-L extraction conditions, for six hop varieties.

## References

[1] Ruano-Rosa D., Garita-Cambronero J. (2023). Microbial Secondary Metabolites in Plant Health. The Chemical Dialogue between Plants and Beneficial Microorganisms.

[2] Veiga B.A., Hamerski F., Clausen M.P., Errico M., de Paula Scheer A., Corazza M.L. (2021). Compressed Fluids Extraction Methods, Yields, Antioxidant Activities, Total Phenolics and Flavonoids Content for Brazilian Mantiqueira Hops. J. Supercrit. Fluids.

[3] Porteous-Álvarez A.J., Maldonado-González M.M., Mayo-Prieto S., Lorenzana A., Paniagua-García A.I., Casquero P.A. (2021). Green Strategies of Powdery Mildew Control in Hop: From Organic Products to Nanoscale Carriers. J. Fungi.

[4] Olšovská J., Kameník Z., Čejka P., Jurková M., Mikyška A. (2013). Ultra-High-Performance Liquid Chromatography Profiling Method for Chemical Screening of Proanthocyanidins in Czech Hops. Talanta.

[5] Sanz V., Torres M.D., López Vilariño J.M., Domínguez H. (2019). What Is New on the Hop Extraction?. Trends Food Sci. Technol..

[6] Almaguer C., Schönberger C., Gastl M., Arendt E.K., Becker T. (2014). *Humulus lupulus*—A Story That Begs to Be Told. A Review. J. Inst. Brew..

[7] Wongchum N., Dechakhamphu A. (2021). Xanthohumol Prolongs Lifespan and Decreases Stress-Induced Mortality in *Drosophila melanogaster*. Comp. Biochem. Physiol. Part C Toxicol. Pharmacol..

[8] Sommella E., Pagano F., Salviati E., Chieppa M., Bertamino A., Manfra M., Sala M., Novellino E., Campiglia P. (2018). Chemical Profiling of Bioactive Constituents in Hop Cones and Pellets Extracts by Online Comprehensive Two-Dimensional Liquid Chromatography with Tandem Mass Spectrometry and Direct Infusion Fourier Transform Ion Cyclotron Resonance Mass Spectrometry. J. Sep. Sci..

[9] Carbone K., Macchioni V., Petrella G., Cicero D.O. (2020). Exploring the Potential of Microwaves and Ultrasounds in the Green Extraction of Bioactive Compounds from *Humulus lupulus* for the Food and Pharmaceutical Industry. Ind. Crop. Prod..

[10] The European Parliament and the Council of The European Union (2009). Directive 2009/32/Ec. Off. J. Eur. Union.

[11] Lorbeer A.J., Lahnstein J., Bulone V., Nguyen T., Zhang W. (2015). Multiple-Response Optimization of the Acidic Treatment of the Brown Alga Ecklonia Radiata for the Sequential Extraction of Fucoidan and Alginate. Bioresour. Technol..

[12] Hrnčič M.K., Španinger E., Košir I.J., Knez Ž., Bren U. (2019). Hop Compounds: Extraction Techniques, Chemical Analyses, Antioxidative, Antimicrobial, and Anticarcinogenic Effects. Nutrients.

[13] Kontek B., Jedrejek D., Oleszek W., Olas B. (2021). Antiradical and Antioxidant Activity in Vitro of Hops-Derived Extracts Rich in Bitter Acids and Xanthohumol. Ind. Crop. Prod..

[14] Lyu J.I., Ryu J., Seo K.S., Kang K.Y., Park S.H., Ha T.H., Ahn J.W., Kang S.Y. (2022). Comparative Study on Phenolic Compounds and Antioxidant Activities of Hop (*Humulus lupulus* L.) Strobile Extracts. Plants.

[15] EBC, E.B.C. 7.2 Moisture Content of Hops and Hop. 1997, 7–9. https://brewup.eu/ebc-analytica/hops-and-hop-products/moisture-content-of-hops-and-hop-products/7.2.

[16] EBC, E.B.C. 7.10 Hop Oil Content of Hops and Hop. **2002**, *1987*, 3–6. https://brewup.eu/ebc-analytica/hops-and-hop-products/hop-oil-content-of-hops-and-hop-products/7.10.

[17] EBC, E.B.C. 7.12 Hop Essential Oils by Capillary Gas Chromatography Flame Ionization Detection. 2006, 7–10. https://brewup.eu/ebc-analytica/hops-and-hop-products/hop-essential-oils-by-capillary-gas-chromatography-flame-ionization-detection/7.12.

[18] EBC, E.B.C. 7.7 A and Β Acids in Hops and Hop Products by HPLC. 2012, 6–11. https://brewup.eu/ebc-analytica/hops-and-hop-products/and-acids-in-hops-and-hop-products-by-hplc7/7.7.

[19] EBC, E.B.C. 7.15 Xanthohumol in Hops and Hop Products By Hplc (Vm). 2017, *7*, 1–5. https://brewup.eu/ebc-analytica/hops-and-hop-products/xanthohumol-in-hops-and-hop-products-by-hplc/7.15.

[20] EBC, E.B.C. 7.14 Total Polyphenols in Hops and Hop. 2015, *11*, 7–10. https://brewup.eu/ebc-analytica/hops-and-hop-products/total-polyphenols-in-hops-and-hop-pellets/7.14.

[21] Grudniewska A., Popłoński J. (2020). Simple and Green Method for the Extraction of Xanthohumol from Spent Hops Using Deep Eutectic Solvents. Sep. Purif. Technol..

[22] Singleton V.L., Rossi J.A. (1965). Colorimetry of Total Phenolics with Phosphomolybdic-Phosphotungstic Acid Reagents. Am. J. Enol. Vitic..

[23] Chiancone B., Guarrasi V., Leto L., Del Vecchio L., Calani L., Ganino T., Galaverni M., Cirlini M. (2023). Vitro-Derived Hop (*Humulus lupulus* L.) Leaves and Roots as Source of Bioactive Compounds: Antioxidant Activity and Polyphenolic Profile. Plant Cell Tissue Organ Cult..

[24] Squillaci G., Apone F., Sena L.M., Carola A., Tito A., Bimonte M., De Lucia A., Colucci G., La Cara F., Morana A. (2018). Chestnut (*Castanea sativa* Mill.) Industrial Wastes as a Valued Bioresource for the Production of Active Ingredients. Process Biochem..

[25] Chadwick L.R., Pauli G.F., Farnsworth N.R. (2006). The Pharmacognosy of *Humulus lupulus* L. (Hops) with an Emphasis on Estrogenic Properties. Phytomedicine.

[26] Zanoli P., Zavatti M. (2008). Pharmacognostic and Pharmacological Profile of *Humulus lupulus* L. J. Ethnopharmacol..

[27] Proestos C., Komaitis M. (2008). Antioxidant Capacity of Hops.

[28] Inui T., Okumura K., Matsui H., Hosoya T., Kumazawa S. (2017). Effect of Harvest Time on Some in Vitro Functional Properties of Hop Polyphenols. Food Chem..

[29] Kim E.M., Eom J.H., Um Y., Kim Y., Woo H.M. (2015). Microbial Synthesis of Myrcene by Metabolically Engineered *Escherichia coli*. J. Agric. Food Chem..

[30] Dresel M., Dunkel A., Hofmann T. (2015). Sensomics Analysis of Key Bitter Compounds in the Hard Resin of Hops (*Humulus lupulus* L.) and Their Contribution to the Bitter Profile of Pilsner-Type Beer. J. Agric. Food Chem..

[31] Taniguchi Y., Taniguchi H., Yamada M., Matsukura Y., Koizumi H., Furihata K., Shindo K. (2014). Analysis of the Components of Hard Resin in Hops (*Humulus lupulus* L.) and Structural Elucidation of Their Transformation Products Formed during the Brewing Process. J. Agric. Food Chem..

[32] Stevens J.F., Page J.E. (2004). Xanthohumol and Related Prenylflavonoids from Hops and Beer: To Your Good Health!. Phytochemistry.

[33] Önder F.C., Ay M., Sarker S.D. (2013). Comparative Study of Antioxidant Properties and Total Phenolic Content of the Extracts of *Humulus lupulus* L. and Quantification of Bioactive Components by LC-MS/MS and GC-MS. J. Agric. Food Chem..

[34] Kowalczyk D., Świeca M., Cichocka J., Gawlik-Dziki U. (2013). The Phenolic Content and Antioxidant Activity of the Aqueous and Hydroalcoholic Extracts of Hops and Their Pellets. J. Inst. Brew..

[35] Keskin, Şirin Y., Çakir H.E., Keskin M. (2019). An Investigation of *Humulus lupulus* L.: Phenolic Composition, Antioxidant Capacity and Inhibition Properties of Clinically Important Enzymes. S. Afr. J. Bot..

[36] Piechowiak T., Grzelak-Błaszczyk K., Bonikowski R., Balawejder M. (2020). Optimization of Extraction Process of Antioxidant Compounds from Yellow Onion Skin and Their Use in Functional Bread Production. LWT.

[37] Silva E.M., Rogez H., Larondelle Y. (2007). Optimization of Extraction of Phenolics from Inga Edulis Leaves Using Response Surface Methodology. Sep. Purif. Technol..

[38] Vázquez G., Fernández-Agulló A., Gómez-Castro C., Freire M.S., Antorrena G., González-Álvarez J. (2012). Response Surface Optimization of Antioxidants Extraction from Chestnut (*Castanea sativa*) Bur. Ind. Crop. Prod..

[39] Lakka A., Karageorgou I., Kaltsa O., Batra G., Bozinou E., Lalas S., Makris D. (2019). Polyphenol Extraction from *Humulus lupulus* (Hop) Using a Neoteric Glycerol/L-Alanine Deep Eutectic Solvent: Optimisation, Kinetics and the Effect of Ultrasound-Assisted Pretreatment. AgriEngineering.

[40] Nagybákay N.E., Syrpas M., Vilimaitė V., Tamkutė L., Pukalskas A., Venskutonis P.R., Kitrytė V. (2021). Optimized Supercritical CO_2_ Extraction Enhances the Recovery of Valuable Lipophilic Antioxidants and Other Constituents from Dual-Purpose Hop (*Humulus lupulus* L.) Variety Ella. Antioxidants.

[41] Almeida A.d.R., Maciel M.V.d.O.B., Machado M.H., Bazzo G.C., de Armas R.D., Vitorino V.B., Vitali L., Block J.M., Barreto P.L.M. (2020). Bioactive Compounds and Antioxidant Activities of Brazilian Hop (*Humulus lupulus* L.) Extracts. Int. J. Food Sci. Technol..

[42] Arruda T.R., Pinheiro P.F., Silva P.I., Bernardes P.C. (2021). A New Perspective of a Well-Recognized Raw Material: Phenolic Content, Antioxidant and Antimicrobial Activities and α- and β-Acids Profile of Brazilian Hop (*Humulus lupulus* L.) Extracts. LWT.

[43] Labieniec-Watala M., Przygodzki T., Siewiera K., Podsedek A., Watala C. (2015). Facts and Artifacts in the Evaluation of the Anti-Diabetic Activity of Spent Hop Extract in Rat Hearts in the “Experimental Model of Diabetes”. Int. J. Pharm. Sci. Res..

[44] Ting P.L., Lusk L., Refling J., Kay S., Ryder D. (2008). Identification of Antiradical Hop Compounds. J. Am. Soc. Brew. Chem..

[45] Yamaguchi N., Satoh-Yamaguchi K., Ono M. (2009). In Vitro Evaluation of Antibacterial, Anticollagenase, and Antioxidant Activities of Hop Components (*Humulus lupulus*) Addressing Acne Vulgaris. Phytomedicine.

