# Supplementary material for: Fractionation of High-Value Compounds from Hops Using an Optimised Sequential Extraction Procedure
Source: Antioxidants (Basel). 2023 Dec 26;13(1):45. doi: 10.3390/antiox13010045 (PMC10812624; doi:10.3390/antiox13010045)
Supplement: Supplementary file 1 [file antioxidants-13-00045-s001.zip › antioxidants-2739749-supplementary.pdf]

Supplementary Materials

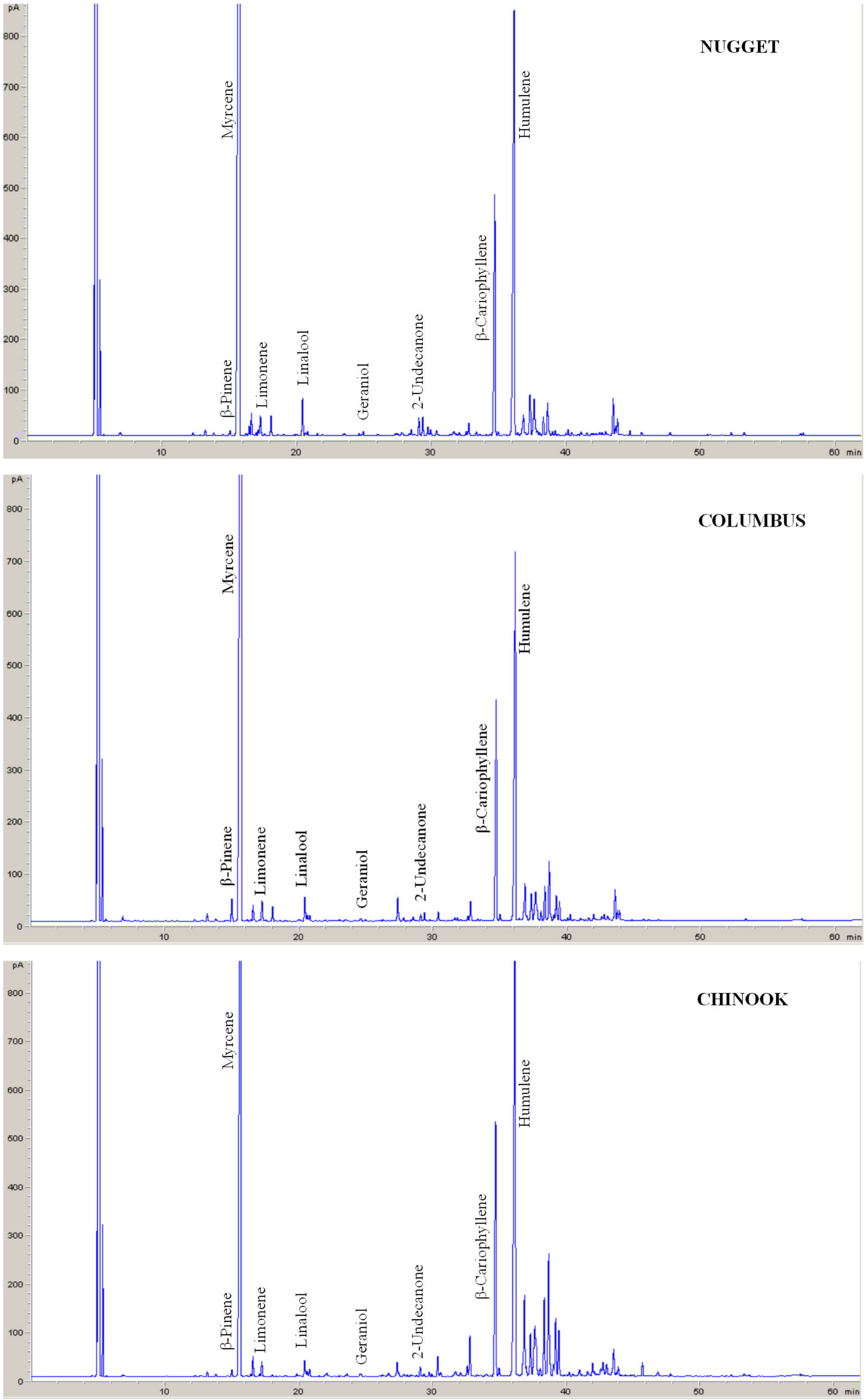

Figure S1. (Continued).

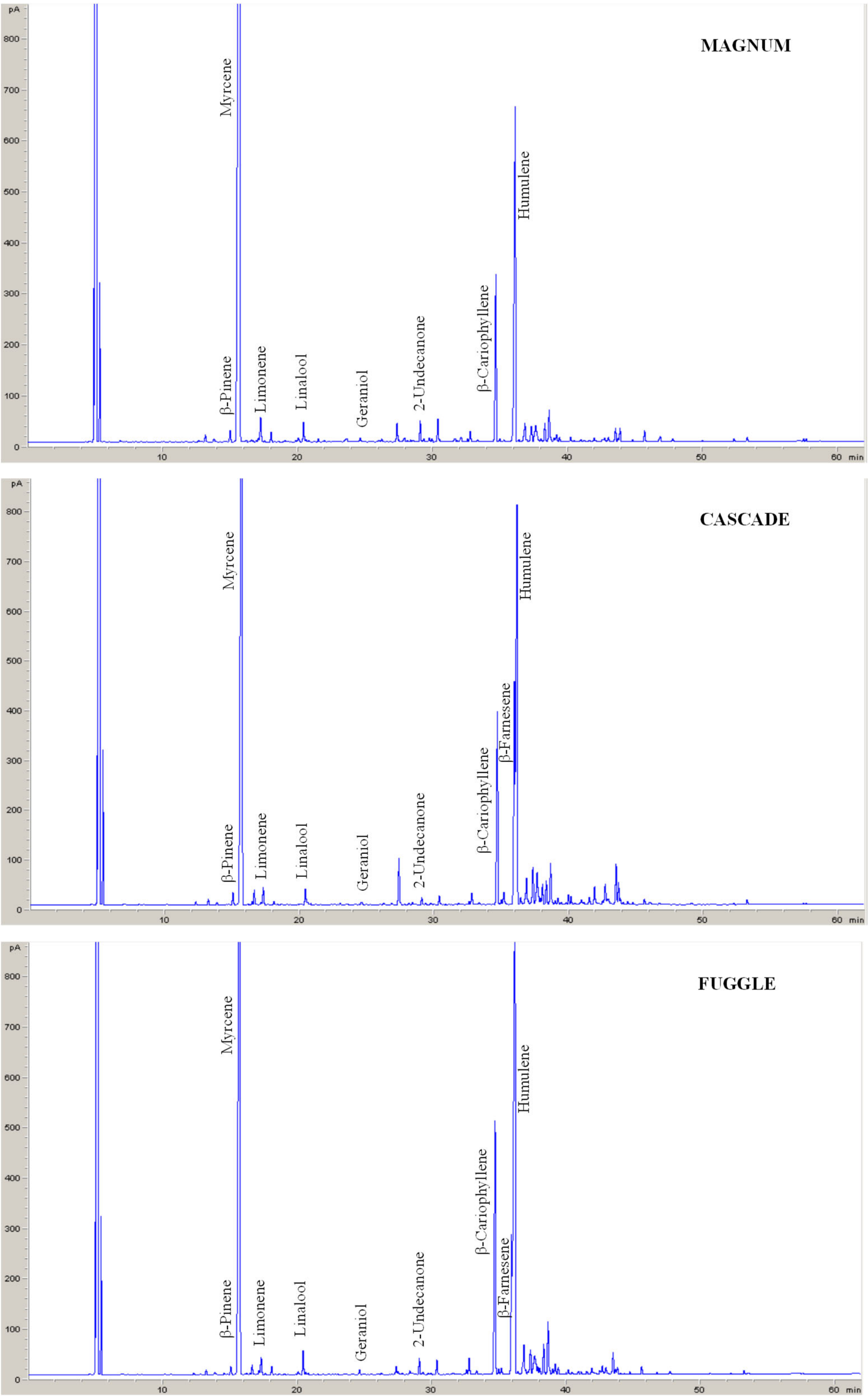

Figure S1. GC chromatograms of the essential oils obtained from the six hop varieties (Nugget, Columbus, Chinook, Magnum, Cascade and Fuggle).

Table S2. Experimental results of the recoveries (%) of α- and β-acids, xanthohumol and total phenolic compounds (TPC) in soft resins, hard resins and spent solids according to experimental design.

Trial	Methanol concentration % (v/v)	Stirring time (min)	α-Acids Yield (%)	β-Acids Yield (%)	Xanthohumol Yield (%)	TPC Yield (%)
Soft resins						
1	25	62.5	88.03	92.50	8.35	na
2	25	62.5	78.59	82.66	8.13	na
3	50	62.5	70.43	82.45	7.04	na
4	25	62.5	81.23	86.06	8.55	na
5	25	62.5	86.74	90.91	6.72	na
6	25	120	87.99	91.50	8.61	na
7	7	21.8	79.81	82.24	7.11	na
8	25	5.0	75.96	86.46	6.68	na
9	43	103.2	71.90	79.71	7.44	na
10	0	62.5	87.45	87.16	7.04	na
11	25	62.5	81.89	85.61	6.87	na
12	43	21.8	71.31	81.29	6.51	na
13	7	103.2	83.91	83.00	6.83	na
Hard resins						
1	25	62.5	8.85	3.05	80.28	14.54
2	25	62.5	8.80	3.52	78.48	13.53
3	50	62.5	16.74	7.46	78.01	26.28
4	25	62.5	8.77	3.09	79.20	13.54
5	25	62.5	7.38	2.57	79.96	10.70
6	25	120	6.25	1.81	78.69	12.14
7	7	21.8	5.13	2.85	74.43	9.70
8	25	5.0	12.12	3.46	77.07	11.89
9	43	103.2	12.92	5.99	78.65	20.36
10	0	62.5	2.57	1.58	65.47	3.79
11	25	62.5	7.11	2.39	77.05	13.33
12	43	21.8	13.70	7.13	77.56	20.74
13	7	103.2	4.56	2.73	77.71	13.41
Spent solids						
1	25	62.5	1.64	0.51	1.90	65.86
2	25	62.5	1.96	0.77	2.15	67.76
3	50	62.5	1.90	0.66	1.44	61.89
4	25	62.5	2.25	0.93	2.28	65.42
5	25	62.5	1.65	0.61	2.05	65.52
6	25	120	2.21	0.96	2.00	66.45
7	7	21.8	3.07	1.47	4.81	76.27
8	25	5.0	2.82	0.96	4.66	69.47
9	43	103.2	1.69	0.61	1.35	59.76
10	0	62.5	2.49	0.65	13.40	73.58
11	25	62.5	2.56	1.15	2.42	69.22
12	43	21.8	2.85	1.27	2.91	65.77
13	7	103.2	1.56	0.56	1.93	69.83

na: Not analysed

Table S3. Estimated regression coefficients for the recovery of α-acids in soft resins. Note: this model includes all the terms.

Term	Coefficient	Standard error coef.	T	p
Constant	74.136	5.651	13.119	0.000
Methanol (%)	0.325	0.270	1.202	0.268
Time (min)	0.249	0.121	2.056	0.079
Methanol (%) * Methanol (%)	-0.011	0.004	-2.657	0.033
Time (min) * Time (min)	-0.012	0.001	-1.515	0.174
Methanol (%) * Time (min)	-0.012	0.002	-0.502	0.631
S = 3.498050	PRESS=401.992			
R-square = 82.84%	R-square (pred) = 19.50%	R-square (adj) = 83.29%		

Table S4. Estimated regression coefficients for the recovery of β-acids in soft resins. Note: this model includes all the terms.

Term	Coefficient	Standard error coef.	T	p
Constant	78.453	6.167	12.721	0.000
Methanol (%)	0.520	0.295	1.762	0.121
Time (min)	0.140	0.132	1.057	0.325
Methanol (%) * Methanol (%)	-0.011	0.004	-2.357	0.051
Time (min) * Time (min)	-0.001	0.001	-0.915	0.391
Methanol (%) * Time (min)	-0.001	0.003	-0.306	0.768
S = 3.81792	PRESS=522.733			
R-square = 51.34%	R-square (pred) = 0.00%	R-square (adj) = 16.59%		

Table S5. Estimated regression coefficients for the recovery of xanthohumol in hard resins. Note: this model includes all the terms.

Term	Coefficient	Standard error coef.	T	p
Constant	66.700	3.936	16.946	0.000
Methanol (%)	0.683	0.188	3.629	0.008
Time (min)	0.335	0.084	0.414	0.692
Methanol (%) * Methanol (%)	-0.010	0.003	-3.258	0.014
Time (min) * Time (min)	0.000	0.001	0.066	0.949
Methanol (%) * Time (min)	-0.001	0.002	-0.450	0.667
S = 2.43682	PRESS=254.801			
R-square = 75.85%	R-square (pred) = 0.00%	R-square (adj) = 58.60%		

Table S6. Estimated regression coefficients for the recovery of total phenolic compounds (TPC) in spent solids. Note: this model includes all the terms.

Term	Coefficient	Standard error coef.	T	p
Constant	79.205	2.851	27.779	0.000
Methanol (%)	-0.351	0.136	-2.572	0.037
Time (min)	-0.101	0.061	-1.652	0.142
Methanol (%) * Methanol (%)	0.002	0.002	0.739	0.484
Time (min) * Time (min)	0.000	0.000	0.907	0.395
Methanol (%) * Time (min)	0.000	0.001	0.122	0.906
S = 1.76518	PRESS=91.8260			
R-square = 90.63%	R-square (pred) = 60.53%	R-square (adj) = 83.93%		

Table S7. Analysis of variance for the recovery of α-acids in soft resins. Note: this model includes all the terms.

Source	df	Sum of squares Seq.	Sum of squares Adjust.	Mean squares Adjust.	F	p
Regression	5	413.70	413.70	82.74	6.76	0.013
Lineal	2	307.25	54.56	27.28	2.23	0.178
Methanol (%)	1	248.39	17.68	17.68	1.44	0.268
Time (min)	1	58.87	51.73	51.73	4.23	0.079
Quadratic	2	103.37	103.37	51.68	4.22	0.063
Methanol (%) * Methanol (%)	1	75.29	86.38	86.38	7.06	0.033
Time (min) * Time (min)	1	28.08	28.08	28.08	2.29	0.174
Interaction	1	3.08	3.08	3.08	0.25	0.631
Methanol (%) * Time (min)	1	3.08	3.08	3.08	0.25	0.631
Residual error	7	85.68	85.68	12.24		
Lack of fit	3	48.32	48.32	16.11	1.72	0.299
Pure error	4	37.35	37.35	9.34		
Total	12	499.38				

Table S8. Analysis of variance for the recovery of β-acids in soft resins. Note: this model includes all the terms.

Source	df	Sum of squares Seq.	Sum of squares Adjust.	Mean squares Adjust.	F	p
Regression	5	107.67	107.67	21.53	1.48	0.308
Lineal	2	19.82	48.09	24.04	1.65	0.259
Methanol (%)	1	14.86	45.25	45.25	3.10	0.121
Time (min)	1	4.96	16.30	16.30	1.12	0.325
Quadratic	2	86.48	86.48	43.24	2.97	0.117
Methanol (%) * Methanol (%)	1	74.28	81.01	81.01	5.56	0.051
Time (min) * Time (min)	1	12.20	12.20	12.20	0.84	0.391
Interaction	1	1.37	1.37	1.37	0.09	0.768
Methanol (%) * Time (min)	1	1.37	1.37	1.37	0.09	0.768
Residual error	7	102.04	102.04	14.58		
Lack of fit	3	65.48	65.48	21.825	2.39	0.210
Pure error	4	36.56	36.56	9.14		
Total	12	209.70				

Table S9. Analysis of variance for the recovery of xanthohumol in hard resins. Note: this model includes all the terms.

Source	df	Sum of squares Seq.	Sum of squares Adjust.	Mean squares Adjust.	F	p
Regression	5	130.57	130.57	26.11	4.40	0.039
Lineal	2	64.89	84.00	42.00	7.07	0.021
Methanol (%)	1	59.35	78.20	78.20	13.17	0.008
Time (min)	1	5.55	1.016	1.016	0.17	0.692
Quadratic	2	64.47	64.47	32.24	5.43	0.038
Methanol (%) * Methanol (%)	1	64.45	63.02	63.02	10.61	0.014
Time (min) * Time (min)	1	0.03	0.03	0.03	0.00	0.949
Interaction	1	1.20	1.20	1.20	0.20	0.667
Methanol (%) * Time (min)	1	1.20	1.20	1.20	0.20	0.667
Residual error	7	41.57	41.57	5.94		
Lack of fit	3	34.22	34.22	11.41	6.21	0.055
Pure error	4	7.35	7.35	1.84		
Total	12	172.13				

Table S10. Analysis of variance for the recovery of total phenolic compounds (TPC) in spent solids. Note: this model includes all the terms.

Source	df	Sum of squares Seq.	Sum of squares Adjust.	Mean squares Adjust.	F	p
Regression	5	210.85	210.85	42.17	13.53	0.002
Lineal	2	207.02	22.42	11.21	3.60	0.084
Methanol (%)	1	172.07	20.61	20.61	6.62	0.037
Time (min)	1	34.95	8.51	8.51	2.73	0.142
Quadratic	2	3.78	3.78	1.89	0.61	0.571
Methanol (%) * Methanol (%)	1	1.22	1.70	1.70	0.55	0.484
Time (min) * Time (min)	1	2.56	2.56	2.56	0.82	0.395
Interaction	1	0.05	0.05	0.05	0.01	0.906
Methanol (%) * Time (min)	1	0.05	0.05	0.05	0.01	0.906
Residual error	7	21.81	21.81	3.12		
Lack of fit	3	10.41	10.41	3.47	1.22	0.412
Pure error	4	11.40	11.40	2.851		
Total	12	232.66				

Table S11. Weight and chemical composition (α- and β-acids, xanthohumol and total phenolic compounds (TPC)) of the initial hop pellets and the fractions obtained after the sequential extraction process carried out under optimal S-L extraction conditions, for six hop varieties.

	WEIGHT (g)	Concentration (g/100 g)							
		Cohumulone	n+Adhumulone	Colupulone	n+Adlupulone	α-Acids	β-Acids	Xanthohumol	TPC (*)
				Nugget					
Initial Hops	*20.00*	*2.28 ± 0.04*	*7.98 ± 0.08*	*1.82 ± 0.02*	*1.83 ± 0.02*	*10.26 ± 0.12*	*3.65 ± 0.04*	*0.68 ± 0.01*	*2.10 ± 0.02*
Soft Resins	4.15 ± 0.18	8.93 ± 0.29	30.26 ± 0.76	7.51 ± 0.37	7.53 ± 0.25	39.19 ± 1.03	15.05 ± 0.58	0.21 ± 0.00	na
Hard Resins	1.96 ± 0.07	1.56 ± 0.08	5.38 ± 0.31	0.48 ± 0.02	0.41 ± 0.04	6.94 ± 0.40	0.89 ± 0.05	5.56 ± 0.25	3.07 ± 0.15
Spent Solid	14.33 ± 0.08	0.08 ± 0.03	0.27 ± 0.10	0.03 ± 0.01	0.03 ± 0.01	0.36 ± 0.13	0.06 ± 0.02	0.02 ± 0.01	1.97 ± 0.04
				Columbus					
Initial Hops	*20.00*	*3.51 ± 0.05*	*8.45 ± 0.08*	*2.29 ± 0.00*	*1.84 ± 0.02*	*11.96 ± 0.13*	*4.13 ± 0.02*	*0.70 ± 0.01*	*1.75 ± 0.03*
Soft Resins	3.92 ± 0.22	11.13 ± 0.41	27.23 ± 1.07	8.37 ± 0.30	6.92 ± 0.24	38.37 ± 1.47	15.29 ± 0.54	0.23 ± 0.00	na
Hard Resins	1.64 ± 0.07	3.53 ± 0.26	8.86 ± 0.96	0.81 ± 0.14	0.64 ± 0.16	12.39 ± 1.21	1.45 ± 0.30	5.61 ± 0.14	3.31 ± 0.23
Spent Solid	14.57 ± 0.06	0.43 ± 0.02	1.02 ± 0.05	0.24 ± 0.02	0.20 ± 0.01	1.45 ± 0.08	0.43 ± 0.03	0.08 ± 0.01	1.75 ± 0.04
				Chinook					
Initial Hops	*20.00*	*2.80 ± 0.12*	*6.22 ± 0.18*	*1.46 ± 0.06*	*1.13 ± 0.03*	*9.02 ± 0.29*	*2.58 ± 0.09*	*0.55 ± 0.01*	*2.75 ± 0.03*
Soft Resins	3.30 ± 0.13	13.03 ± 0.90	30.18 ± 1.94	7.43 ± 0.32	5.89 ± 0.23	43.22 ± 2.84	13.31 ± 0.54	0.36 ± 0.03	na
Hard Resins	1.19 ± 0.42	1.69 ± 0.23	3.85 ± 0.70	0.46 ± 0.08	0.52 ± 0.05	5.54 ± 0.93	0.98 ± 0.04	7.36 ± 2.93	4.29 ± 0.68
Spent Solid	15.03 ± 0.25	0.12 ± 0.02	0.25 ± 0.04	0.04 ± 0.01	0.03 ± 0.01	0.37 ± 0.06	0.07 ± 0.02	0.02 ± 0.00	2.94 ± 0.09
				Magnum					
Initial Hops	*20.00*	*2.00 ± 0.01*	*4.84 ± 0.07*	*1.48 ± 0.02*	*1.14 ± 0.02*	*6.84 ± 0.08*	*2.62 ± 0.03*	*0.53 ± 0.01*	*2.81 ± 0.02*
Soft Resins	2.76 ± 0.05	12.12 ± 0.23	29.77 ± 0.41	9.57 ± 0.21	7.44 ± 0.10	41.89 ± 0.64	17.01 ± 0.31	0.67 ± 0.01	na
Hard Resins	0.85 ± 0.07	1.86 ± 0.20	5.10 ± 0.66	0.86 ± 0.06	0.82 ± 0.05	6.96 ± 0.86	1.68 ± 0.10	9.13 ± 0.74	5.24 ± 0.49
Spent Solid	15.73 ± 0.06	0.07 ± 0.01	0.16 ± 0.04	0.03 ± 0.01	0.02 ± 0.01	0.23 ± 0.05	0.05 ± 0.02	0.02 ± 0.00	2.78 ± 0.07
				Cascade					
Initial Hops	*20.00*	*1.59 ± 0.01*	*3.09 ± 0.01*	*2.30 ± 0.02*	*2.28 ± 0.03*	*4.68 ± 0.01*	*4.58 ± 0.04*	*0.33 ± 0.00*	*2.75 ± 0.01*
Soft Resins	2.76 ± 0.02	8.92 ± 0.12	17.62 ± 0.26	14.77 ± 0.45	14.52 ± 0.34	26.54 ± 0.38	29.29 ± 0.78	0.51 ± 0.02	na
Hard Resins	1.10 ± 0.20	2.37 ± 0.20	4.42 ± 0.29	0.99 ± 0.22	0.99 ± 0.27	6.79 ± 0.49	1.98 ± 0.48	4.28 ± 0.88	5.93 ± 0.68
Spent Solid	15.77 ± 0.12	0.07 ± 0.01	0.12 ± 0.01	0.04 ± 0.01	0.04 ± 0.01	0.19 ± 0.02	0.08 ± 0.01	0.01 ± 0.00	2.74 ± 0.03
				Fuggle					
Initial Hops	*20.00*	*1.59 ± 0.06*	*4.71 ± 0.04*	*1.40 ± 0.02*	*1.59 ± 0.02*	*6.30 ± 0.10*	*2.99 ± 0.03*	*0.40 ± 0.00*	*3.28 ± 0.03*
Soft Resins	3.30 ± 0.54	7.41 ± 1.10	23.01 ± 2.65	7.54 ± 1.04	8.61 ± 0.97	30.42 ± 3.71	16.15 ± 1.98	0.57 ± 0.11	na
Hard Resins	1.01 ± 0.13	1.88 ± 0.40	6.74 ± 2.25	0.91 ± 0.40	1.12 ± 0.41	8.62 ± 2.63	2.04 ± 0.81	5.71 ± 0.82	4.73 ± 0.71
Spent Solid	15.97 ± 0.15	0.05 ± 0.01	0.14 ± 0.03	0.02 ± 0.01	0.02 ± 0.01	0.19 ± 0.04	0.05 ± 0.01	0.01 ± 0.00	3.56 ± 0.17

(*) g gallic acid equivalents/100 (g GAE/100 g)
na: Not analysed
